# Whole-genome sequencing of wild Siberian musk deer (*Moschus moschiferus*) provides insights into its genetic features

**DOI:** 10.1186/s12864-020-6495-2

**Published:** 2020-01-31

**Authors:** Li Yi, Menggen Dalai, Rina Su, Weili Lin, Myagmarsuren Erdenedalai, Batkhuu Luvsantseren, Chimedragchaa Chimedtseren, Zhen Wang, Surong Hasi

**Affiliations:** 1 Inner Mongolia Agricultural University Key /Laboratory of Clinical Diagnosis and Treatment Technology in Animal Disease, Ministry of Agriculture and Rural Affairs , Hohhot, 010018 China; 2 Affiliated Hospital of Inner Mongolia Medical University, Hohhot, 010050 China; 3 Key Laboratory of Computational Biology, CAS-MPG Partner Institute for Computational Biology, Shanghai Institute of Nutrition and Health, Shanghai Institutes for Biological Sciences, Chinese Academy of Sciences, Shanghai, 200031 China; 4 Institute of Traditional Medicine and Technology, Ulaanbaatar, Mongolia

**Keywords:** Wild Siberian musk deer (*Moschus moschiferus)* genome, De novo assembly, Genetic features, Musk secretion

## Abstract

**Background:**

Siberian musk deer, one of the seven species, is distributed in coniferous forests of Asia. Worldwide, the population size of Siberian musk deer is threatened by severe illegal poaching for commercially valuable musk and meat, habitat losses, and forest fire. At present, this species is categorized as Vulnerable on the IUCN Red List. However, the genetic information of Siberian musk deer is largely unexplored.

**Results:**

Here, we produced 3.10 Gb draft assembly of wild Siberian musk deer with a contig N50 of 29,145 bp and a scaffold N50 of 7,955,248 bp. We annotated 19,363 protein-coding genes and estimated 44.44% of the genome to be repetitive. Our phylogenetic analysis reveals that wild Siberian musk deer is closer to Bovidae than to Cervidae. Comparative analyses showed that the genetic features of Siberian musk deer adapted in cold and high-altitude environments. We sequenced two additional genomes of Siberian musk deer constructed demographic history indicated that changes in effective population size corresponded with recent glacial epochs. Finally, we identified several candidate genes that may play a role in the musk secretion based on transcriptome analysis.

**Conclusions:**

Here, we present a high-quality draft genome of wild Siberian musk deer, which will provide a valuable genetic resource for further investigations of this economically important musk deer.

## Background

Musk deer (*Moschus,* Moschidae) are small hornless Pecora ungulates, occurring commonly at mountains and forests of central Asia, belong to Cetartiodactyla, Ruminantia [1, 2]. At present, musk deer comprise seven species, including Anhui musk deer (*M. anhuiensis*), forest musk deer (*M. berezovskii*), Alpine musk deer (*M. chrysogaster*), black musk deer (*M. fuscus*), Himalayan musk deer (*M. leucogaster*), Kashmir musk deer (*M. cupreus*) and Siberian musk deer (*M. moschiferus*) [3–5]. This species is shy, timid, cautious, sensitive, crepuscular and nocturnal, and likes to be alone and does not live in groups [6, 7]. Musk deer inhabits a fairly fixed area throughout its life and rarely changes [1]. Musk deer are famous for secretion musk from the musk gland (only in males), which with specific odor and color, and appear to serve for attracting the females and mark territory [8–10]. Moreover, its secretion is widely used in traditional medicines and perfume industries since the fifth century, because of its unique fragrance and its significant anti-inflammatory and anti-tumor roles, as well as its effects on the human central nervous and cardio-cerebral-vascular systems [11–15]. The musk is regarded as one of the most valuable of all animal scents, even more, expensive than gold [16]. However, the population of musk deer has dramatically decreased due to illegal poaching for their meat and musk, exploitation of natural resources, trade, infrastructure construction, fast urbanization [16–19]. Therefore, six species being listed as endangered and one as vulnerable by the International Union for Conservation of Nature (IUCN 2017) [20]. All of them are also listed in Category I of the State Key Protected Wildlife List of China [21].

In recent years, there has been significant progress in the studies of musk deer ecology, taxonomy, evolution history by paleontological, morphological, ecological and ethological and molecular analysis [22–40]. The musk composition and secretory mechanism of musk have been explored by various aspects, including microsatellite, mtDNA marker, and transcriptome sequencing data [41–46]. Besides, the gut microbial communities have been illustrated by metagenome sequencing [9, 47, 48]. Unfortunately, genomic resources of the species are rarely limited. Recent work has provided the first complete genome sequence of the forest musk deer [49]. Siberian musk deer is one of the seven species, widely occurs in Korea, Mongolia, Russia, China, Kazakhstan, Kyrgyzstan, Nepal, and Vietnam [50]. However, the population size of Siberian musk deer is dwindling rapidly by the same reasons as other musk species, and they have been categorized as Vulnerable on the IUCN Red List [51]. As a result of the extinction crisis of Siberian musk deer and economic and medical value of its musk, understanding the genetic basis and features, environment adaptions, and the musk secretion mechanism is necessary. However, the whole-genome sequencing of Siberian musk deer has not been performed, and their potential value has yet to be discovered.

In this study, we perform high-quality whole-genome sequencing of three wild Siberian musk deer (WSMD) from Mongolia, and transcriptome sequencing of one mixture of tissue from a naturally died female WSMD. These genomic and transcriptome analyses provide evidence of Siberian musk deer genetic features and musk secretion.

## Results

### Genome sequencing, assembly, and evaluation

Genomic DNA extracted from a female WSMD was subjected to shotgun sequencing using the Illumina Hiseq Xten platform. We prepared 19 pair-end libraries spanning several insert sizes (from 250 bp to 10 kb, Additional file 1: Table S1) to generate short pair-end reads. A total of 326.64 Gb (102.97× coverage) raw data were generated from all constructed libraries, from which 283.22Gb of clean data was obtained after removal of low-quality reads, duplicates, adaptors, and reads with more than 10% N bases. The genome assembly was estimated to be approximately 3.10Gb using K-mer = 41 analysis [52], which was slightly bigger than that of the forest musk deer (2.72Gb) [49]. The assembly consisted of 13,344 scaffolds (≥1 kb) with an N50 of 7,955,248 bp and 165,764 contigs with an N50 of 29,145 bp (Table 1). The genome-wide proportion of G + C was 41.96% (Additional file 1: Table S2). By mapping the short-fragment libraries to the assembled genome with BWA mem (v0.7.12), 98% reads were mappable (93.16% properly paired), indicating a highly accurate assembly (Additional file 1: Table S3).

**Table 1:** Statistics of the genome assembly (The minimum size of contigs for reporting is 1 Kb)

Statistics	Size of contigs (bp)	Size of scaffolds (bp)	Number of contigs	Number of scaffolds
Total	2,435,924,293	2,703,175,379	165,764	13,344
Max	498,578	35,164,634	–	–
N50	29,145	7,955,248	23,516	93
N60	22,936	6,419,411	32,950	130
N70	17,477	4,597,695	45,098	179
N80	12,390	3,185,516	61,590	250
N90	7170	1,717,083	87,009	365

Subsequently, we used Benchmarking Universal Single Copy Orthologs BUSCO (BUSCO, V2.0) [53] to assess the completeness of the genome assembly. BUSCO results showed that 93.30% of the 4104 mammalian single-copy orthologues were complete (Additional file 1: Table S4). Furthermore, we downloaded the musk gland and heart RNA-sequencing data (SRA accession: SRR2098995, SRR2098996, and SRR2142357) of forest musk deer from the National Center for Biotechnology Information (NCBI) and mapped to the genome assembly using STAR [54]. The alignment coverage of expressed sequences was ranged from 35 to 75% in the genome assembly. These assessments indicated that our assembly with a high level of completeness. Hence, a high-quality assembly of WSMD is provided here, rendering it a valuable source for studying genome structure and evolution.

### Genome comparison of Siberian musk deer and forest musk deer

We compared the genome assembly of the Siberian musk deer and forest musk deer recently reported by Fan et al. [55] (Additional file 3: Table S17). The continuity of our assembly was remarkably increased compared with that of the forest musk deer genome assembly, particularly in regard to the scaffold N50 (7.95 vs 2.85 Mb) and scaffold number (13,344 vs 79,206). We then aligned the two genome assemblies using mummer4 [56]. At least 2.16 Gb (80.16%) of our assembly could be aligned with that of the forest musk deer, most of which (2.13 Gb) were one-to-one alignment (Additional file 3: Table S17). The average identify of the alignments was 98.74%, suggesting close relationship between the two species.

### Repetitive sequences and gene annotation

Using a combination of homology-based (Ruminant and mammal) and de novo methods, we identified transposable elements (TEs) and other repetitive elements in the WSMD genome. We estimated 44.44% of our genome to be composed of repetitive elements using a combination of homology-based and de novo approaches (Additional file 1: Table S6). The de novo method identified 38.60% of the genome as repetitive, whereas the homology-based method predicted more (44.27 and 43.67%, respectively). The repeat element landscape of WSMD mostly consists of retrotransposons, including long interspersed elements (LINES), short interspersed elements (SINES) and long terminal repeats (LTRs). Among them, LINES represented the most predominant type of repeat sequences, occupying 30.37% of the genome, while the other repeat elements (SINE and LTR) comprised 4.78 and 4.42%, respectively. DNA transposons were particularly rare, forming only 2.27% of the genome.

Gene annotation of the WSMD genome was conducted using several approaches, including ab initio, homology-based and transcript-based methods (Additional file 1: Table S4, Additional file 1: Table S8, and Table S9). Gene models generated from all the methods were integrated by EVM (EvidenceModeler) to build a consensus gene set for the WSMD genome. The final gene set is a union of a gene predicted by Genewise and supplemented with EVM that removed the genes only predicted by ab initio. In total, 19,363 non-redundant protein-coding genes were annotated in the WSMD genome (Additional file 1: Figure S1 and Table S4), which is less than the predicted gene numbers of forest musk deer (24,352 genes) [49]. The BUSCO evaluation showed that 99.1% of genes were identified as complete and fragmented, with genes that were considered missing in the gene set. The BUSCO results showed that our gene predication was more complete (Additional file 1: Table S4). Alongside this, we also provide the length of genes in Additional file 1: Table S8.

### Evolutionary analysis and phylogeny

**Fig. 1:**
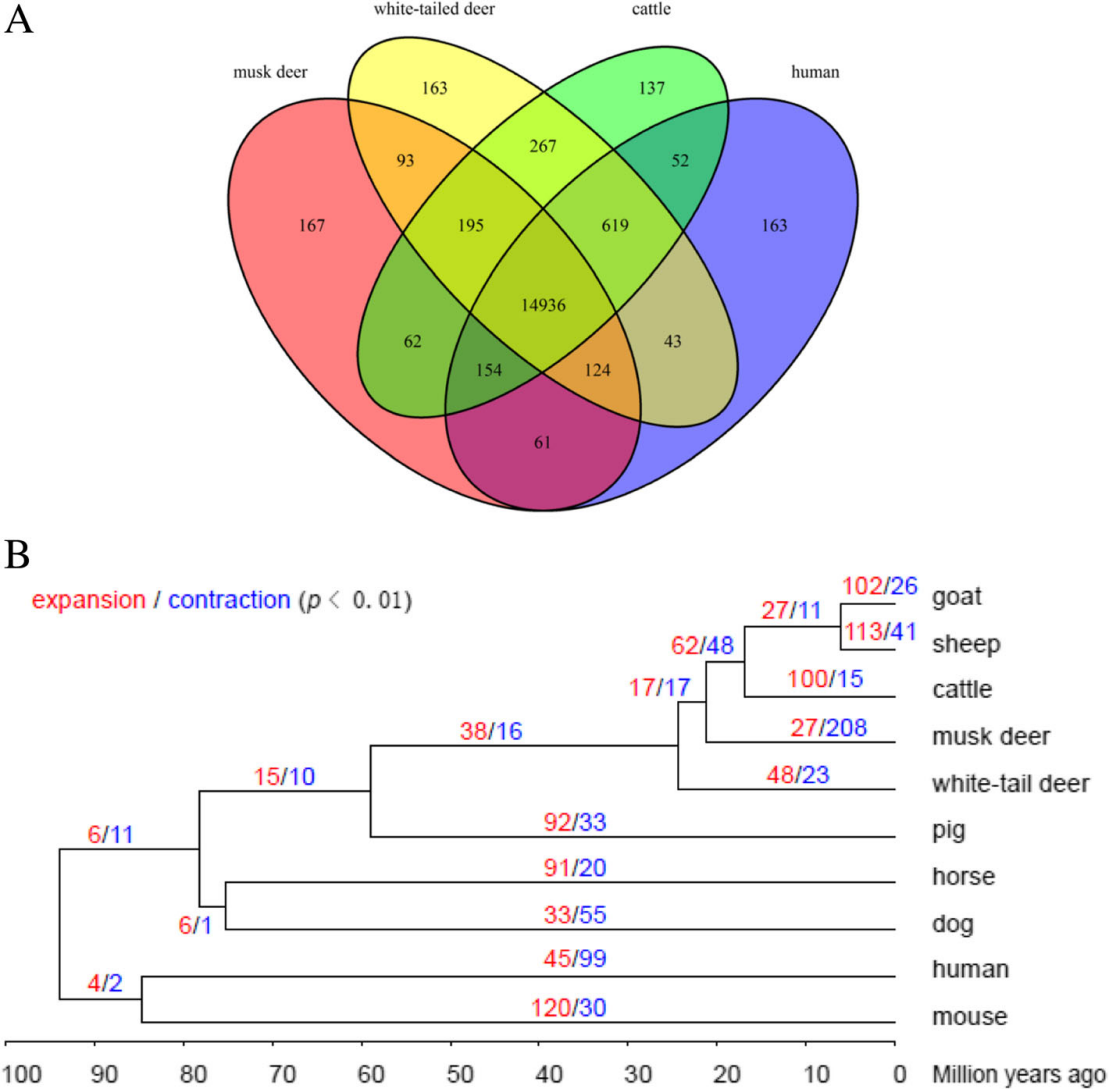
**a** The Venn diagram shows the number of orthologs shared among musk deer and other representative mammals. **b** Phylogeny and gene family size evolution. The phylogenetic tree is constructed based on four-fold degeneration sites among single-copy orthologs with the neighbor-joining method. The timelines indicate inferred divergence times among the species based on the molecular clock. The number of significantly expanded (red) and contracted (blue) gene families (branch-specific *p*-value < 0.01) are shown at each branch

Compared with protein-coding genes of nine other species (goat, sheep, cattle, white-tail deer, pig, horse, dog, human and mouse), we found 17,336 orthologous of WSMD that were shared by at least one species (Additional file 1: Table S11), and 14,936 orthologous shared by human, cattle, white-tailed deer and WSMD. There were 167 gene families specific for WSMD (Fig. 1a). Further, we constructed a phylogenic tree using MEGA based on fourfold degenerate codon sites extracted from single-copy orthologous genes identified by TreeFam (Additional file 1: Table S10 and Fig. 1b). The phylogenic tree was indicated that the WSMD and the Cattle were within a subclade, which was most likely derived from a common ancestor ~ 22 Ma ago (Mya) (Fig. 1b).

Gene gains and losses are one of the primary contributors to functional changes [57]. To obtain greater insight into the evolutionary dynamics of the genes, we determined the expansion and contraction of the gene orthologue clusters among these ten species. We found 27 gene families were expended, whereas 208 gene families were contracted in WSMD (Fig. 1b), which might indicate that losses of function might have an important role in functional evolution. The expanded genes were significantly enriched to several pathways associated with fat digestion and absorption, glycerolipid metabolism, and amino acid metabolism (Additional file 1: Figure S3). The contracted gene families were enriched in pathways related to the sensory system, immune system and infectious diseases (Additional file 1: Figure S4). The corresponding GO terms were shown in Additional file 1: Table S13 and Additional file 1: Table S14.

### Positive selection genes and functional enrichment

To observation of positively selected genes (PSGs) in the WSMD genome raises the question of what signatures of selection are to be found in the extant genomes. A total of 184 PSGs were identified by the branch-site likelihood ratio test, and then mapped them to KEGG pathways and GO categories (Fig. 3b and Additional file 1: Table S15). It was shown that those PSGs are enriched in 8 pathways associated to metabolism (amino sugar and nucleotide sugar metabolism, and lysine degradation), cellular processes (peroxisome and p53 signaling pathway), organismal systems (insulin secretion, pancreatic secretion, mineral absorption and bile secretion), and environmental information processing (cGMP-PKG signaling pathway) (Fig. 3b). GO classification showed that those PSGs are enriched in these functional categories, including cellular components (Cell part, Cell, Intracellular, Intracellular part, Organelle, Membrane-bounded organelle, Cytoplasm, and Intracellular orangelle), biological processes (Cellular process, Single-organism process, single-organism cellular process, and metabolic process) and molecular functions (binding and protein binding)(Additional file 1: Table S15). Musk deer is a nocturnal mammal with sensitive hearing, smell, and sight for its locating food and avoiding predators in darkness [6, 58]. We found 12 PSGs (*ATR, EYA1, NEK4, XRCC1, TRIP12, CNOT8, TOPBP1, PLA2R1, ZFYVE26, UIMC1, MCM10*, and *FBXO18*) were involved in DNA damage and repair categories. This finding possibly avoids the Siberian musk deer from the DNA damage caused by UV radiation and hypoxia in high-altitude environments. Thirty-five PSGs were involved in stress response categories. Among 35 PSGs, 7 genes also associated with the nervous system. In addition, we also observed 2 PSGs (*NR0B2* and *MED25*) distributed in retinoid X receptor binding (GO:0046965, corrected *p*-value = 0.0033).

### Genomic diversity and demography inference

To understand the genetic diversity and demographic history in Siberian musk deer, we sequenced two additional WSMD (one male:s190119001, and one female: s180119002) genome generated a total of 78.27Gb raw data, and for each individual nearly 98% of reads mapped to the reference genome assembly with 8.83× average coverage (Additional file 1: Table S3). We performed single-nucleotide polymorphism (SNP) calling and identified 4.81 million (M) SNPs from three individuals, and the Ts/Tv ratio for SNPs was 1.84 (Additional file 1: Table S11). For each individual, 2,420,974, 2,002,344 and 2,337,725 heterozygous single-nucleotide polymorphisms (SNPs), respectively, along the assembled Siberian musk deer genome (Additional file 1: Table S11).

**Fig. 2:**
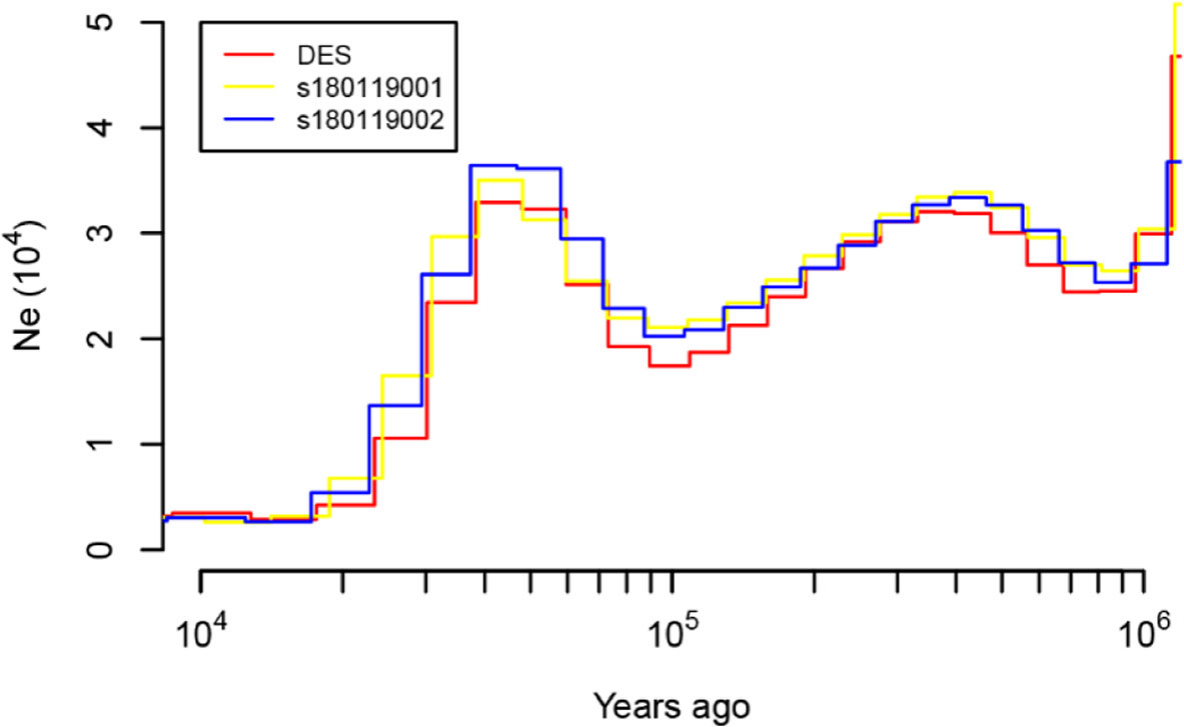
Historical effective population size inferred by PSMC. Each line represents one individual. The result is scaled using a generation time of 5 years and a mutation rate of 1.1 × 10–8 per site per generation

Historical fluctuations in effective population size (*N*_*e*_) for the three individuals were constructed with the help of the Pair-wise Sequentially Markovian Coalescent (PSMC) model [59], three genomes returned concordant PSMC population trajectories that with three declines and two expansions (Fig. 2). The three genomes returned concordant PSMC population trajectories, suggesting no population structure in the species. The first decline in *N*_*e*_ was inferred to have occurred approximately 0.70 Mya, coinciding with the Naynayxungla glaciation (0.78–0.50Mya), which was the most extensive glaciation during the Quaternary Period [60–62]. After the first decline, the *N*_*e*_ for Siberian musk deer recovered and peaked at ~ 0.30 Mya, during the Penultimate glaciation (0.30–0.13 Mya) [60–62]. The cold-climate interval and rising sea level at this stage could have contributed to a population expansion because an increase in grassland was likely under such environmental conditions [63].

The second declines occurring between 0.20 to 0.09 Mya, was detected towards and end of the interglacial period (0.13–0.07 Mya), which presented environmental conditions similar to that of the present [64]. The uplift of the Tibetan Plateau, which caused aridification, and desertification that was dramatically enhanced in the middle Pleistocene age, which reduced the habitat of the musk deer, resulting in a decline of population size [40, 65]. The Siberian musk deer population size then recovered again between 0.05–0.03 Mya during the greatest lake period (0.03–0.04 Mya) because the glaciations were less extended, weather became warm and the forest had expanded that could have contributed to the population expansion [60–62]. Subsequently, a sharp decline in *N*_*e*_ for Siberian musk deer coincided with the extreme cooling climate during the last glaciation (~ 20,000 years ago), it is likely that Siberian musk deer suffered from the effects of climate change, over-hunting, and habitat loss.

### RNA sequencing of mixture tissue

To evaluate the genome completeness, gene annotation and excavating genes related to musk secretion, we sequenced the transcriptome of a mixture tissue (including liver, kidney, lung, heart, skin, and stomach) which collected from a female Siberian musk deer. The Illumina high-throughput next-generation RNA sequencing resulted in 22,927,488 raw reads generated from a mixture of tissue. After removing low-quality sequences, a total of 17,323,786 clean reads were generated. Over 68% of clean reads mapped to the assembly using STAR, suggesting that the majority of transcribed genes are present (Additional file 1: Table S9). After the cufflinks assembly generated 44,271 genes and 61,96 isoforms (Additional file 1: Table S12). Another notable result is that approximately 56% of the counted reads were mapped to exonic regions of a unique gene, and a small proportion of reads (5.8%) were defined as unannotated, which probably contain novel genes and exons (Additional file 1: Table S12).

### Differentially expressed genes and functional enrichment analysis

**Fig. 3:**
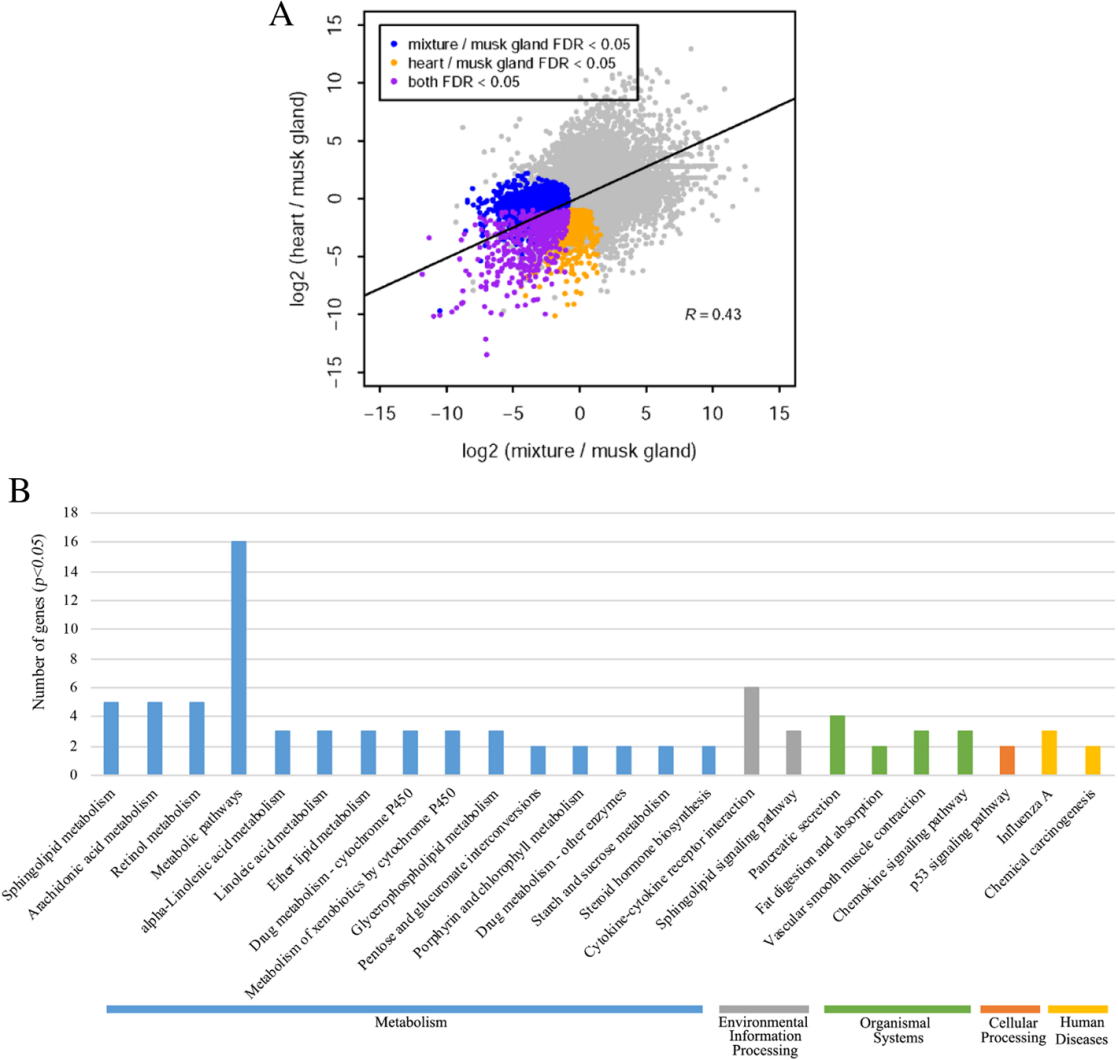
**a** Log_2_-fold change in normalized counts between the mixture tissue and musk gland, as well as between the heart and a musk gland. The points represent genes, and genes with significant over-expression (FDR < 0.05) in the musk gland are colored. A cutoff of log_2_-fold change < − 5 in both comparisons is also applied to screen genes with high expression specifically in the musk gland. **b** KEGG pathway enrichment of DEGs in the Siberian musk deer. The x-axis shows the KEGG functional categories, while eh the number of genes in each category is plotted on the y-axis

We explored the differences among the transcriptomes among the musk gland, heart, and mixture tissue. A total of 189 genes were identified to be upregulated differentially expressed genes (DEGs) in the musk gland, as compared with the same genes in heart and mixture tissues (FDR < 0.05, log_2_-fold change < − 5) (Fig. 3a). There were 78 DEGs that were specifically expressed in the musk gland.

The Go annotation classified the DEGs into 3 categories: molecular functions (MF), cellular components (CC) and biological processes (BP) (Additional file 2: Table S16). Molecular functions included genes mainly involved in binding (112genes, GO:0005488) and protein binding (81genes, GO:0030414). Genes related to cellular components (CC) were primarily cell (136 genes, GO:0005623), cell part (135 genes, GO:0044464), intracellular (117 genes, GO:0005622), intracellular part (112 genes, GO:0044424), organelle (106genes, GO:0043226) and membrane-bounded organelle (102genes, GO:0043227). In addition to the largest proportion of cell-related components, the organelle occupies an important proportion. This result indicates that the molecular components involved in the physiological activities of the siberian musk deer are not only concentrated in cells but also widely distributed in organelles, and play an important role. In the biological process part (BP), a total of 814 terms (7148 genes) are involved, of which the single-organism process (120 genes, GO:0044699) accounts for the largest proportion, followed by metabolic process (98 genes, GO:0008152) and cellular process (118 genes, GO:0008152). Also, it also includes response to the stimulus (71 genes, GO:0050896), cellular response to stimulus (50 genes, GO:0051716), and many categories related to metabolism. This result is consistent with the biological characteristics of the siberian musk deer, which can especially explain its survivability under extreme conditions and its obvious response and alertness to external stimuli [19, 40, 66]. The distribution of GO annotations in different functional categories indicated a substantial diversity of DEGs.

We identified the biochemical pathways based on the DEGs detected in FMD. The KEGG annotation of the DEGs suggested that they were distributed in 24 pathways related to metabolism (59 genes), environmental information processing (9 genes), organismal systems, celluar processing (12 genes), and human diseases (5 genes), (Fig. 3b). Among the identified functional categories of metabolism, metabolic pathways (16 genes) were highly represented, followed by sphingolipid metabolism (5 genes), arachidonic acid metabolism (5 genes), and retinol metabolism (5 genes). In the environmental information processing, mainly has the cytokine-cytokine receotor interaction and sphingolipid signaling pathway. Organismal systems included functions mainly involved in pancreatic secretion, fat digestion and absorption,vascular smooth muscle contraction and chemokine signaling pathway. About human diseases involved in Influenza A and chemical carcinogenesis.

### Genes related to musk secretion

To obtain greater insight into the mechanisms of musk secretion, it was crucial to understanding their metabolic processes and the corresponding pathways and genes. Thus, we screened the GO terms and KEGG pathways associated with the musk compounds and metabolism (Fig. 3b and Additional file 2: Table S16). There were 21 DEGs that were closely involved in related pathways and terms, including steroid biosynthesis and transport (map 00140, GO:0015918 and GO:0036314), terpenoid and diterpenoid metabolic process (GO:0006721 and GO:0016101), hormone response and metabolic process (GO:0009725, GO:0034754, GO:0010817 and GO:0042445), cholesterol transport (GO:0030301) and cytochrome P450 metabolism pathway (map 00980). Among them, *UGT1A4* and *SULT2B1*was annotated in the steroid hormone biosynthesis (map 00140). *UGT1A4* is regarded as the main enzyme that catalyzes N-glucuronidation of various endogenous compounds (eg., steroids and thyroid hormones, fatty acids, bile acids, and bilirubin), as well as of xenobiotics including drugs and foreign compounds [66–68]. *SULT2B1* is a member of the large cytosolic sulfotransferase superfamily that is engaged in the synthesis and metabolism of steroids [69]. It further belongs to the *SULT2* family of enzymes that are primarily involved in the sulfoconjugation of neutral steroids and sterols [70]. It further belongs to the *SULT2* family of enzymes that are primarily involved in the sulfoconjugation of neutral steroids and sterols [70]. Steroid biosynthesis is catalyzed by a suite of enzymes including members of the cytochrome P450 (CYP), short chain dehydrogenase (SDR), and aldo-keto reductase (AKR) superfamilies [71]. CYP2B6, a member of CYP groups of enzyme, was annotated in cytochrome P450 metabolism pathway that participated in the metabolism of arachidonic acid, lauric acid and steroid hormones including testosterone, estrone and 17β-estradiol [72, 73]. It might hint that these genes played significant roles in musk formation and secretion.

## Discussion

In this study, we performed a draft genome of wild Siberian musk deer using next generation sequencing technology. The final assembly of WSMD genome is 3.10 Gb with a contig N50 of 29,145 bp and a scaffold N50 of 7,955,248 bp, accounting for about 87.98% of the whole genome with coverage over 30x. Compared with the genome of the forest musk deer, the present assembly of WSMD has larger genome size, contig N50 and scaffold N50 lengths [49]. The results came from BWA mem, BUSCO and STAR analyses indicated that our assembly with high level of accuracy and completeness, and enough for the following analyses.

We observed that TEs occupied 44.44% of the whole assembly, which was lower than those of cattle (45.14%) and human (46.07%), but larger than those of pig (38.66%), mouse (40.53%) (Additional file 1: Table S7) and forest musk deer (42.05%) [49]. A total of 19,363 non-redundant protein-coding genes was annotated in WSMD genome, which was less than the predicted gene numbers of forest musk deer (24,352 genes) [49]. Moreover, we constructed a phylogenic tree was indicated that the WSMD and the Cattle were within a subclade, which was most likely derived from a common ancestor ~ 22 Ma ago (Mya). Moschidae shows a mixture of Bovidae and Cervidae characteristics [74, 75] so that its phylogenetic status has been strongly debated. The taxonomy of Moschidae as a separate family has been elucidated by the combination of paleontological, morphological, ecological and ethological and molecular analysis [22–32]. However, Moschidea is a sister group of Bovidae or of Cervidae, has obtained different results in different analyses [28, 31–34]. Previous studies on phylogenetic analysis based on whole-genome sequences revealed that forest musk deer as more closely related to Bovidae than to Cervidae, which is consistent with the results of the present study [35, 36, 76]. Historically, the fossil records and some molecular phylogenetic studies regarded Siberian musk deer WSMD as the primitive species in Moschus [25, 37, 38]. However, the divergence time between WSMD and cattle was latter than the time (~ 27.3Mya) at which forest musk deer divided with Bovidae [39]. Pan et al. (2015) have also reported that Siberian musk deer occurs latter than Alpine musk deer branches on the phylogenetic tree based on complete mtDNA analysis [40]. These results were suggested that Siberian musk deer was not the most primitive musk deer.

To adapt to environments of the high mountain forests, Siberian musk deer may have been formed some characteristics under natural selection. It is worth noting that musk deer has sensitive smell and hearing to locating food in darkness. Therefore, it is interesting to uncover evolutionary evidence for its adaptation by comparative analysis. By comparison with nine other species, we found 27 gene families were expended, whereas 208 gene families were contracted in WSMD. Studies have shown that due to the small body size and small appetite musk deer could not get enough food in one time to obtain more energy [77]. Therefore, musk deer often choose high-energy and digestible good, especially in the cold winter and spring when the food is scarce [78]. We found that the expansion gene families were significantly enriched in energy metabolism pathways and GO terms which might help Siberian musk deer to optimize their energy storage and production in the forest. The contraction gene families were most prominent in olfactory transduction pathway (Additional file 1: Figure S4). It might be attributed possibly to musk deer adaptation to the cold and high-altitude environment (1000-4200 m) where food sources and odorants are limited and diffused slowly, and the interactions between odorants and receptors weakened [79, 80]. Similar results have been obtained in some high plateau animal genome studies, such as avian [81], wild boars [82], hot-spring snake [83] and Tibetan chicken [84]. Moreover, we observed 12 PSGs and 2 PSGs were involved in DNA damage and retinoid X receptor binding categories, respectively. These categories seem to be help musk deer living at high altitudes avoid high levels of ultraviolet radiation and forage in darkness. The previous study based on the forest musk genome has identified eight PSGs genes enriched in the phototransduction pathway and retinol metabolism pathways [35]. Our results and theirs did not have overlap candidate genes. Taken together, these results provide evidence for musk deer to adapt to the environments. In addition, the demographic historical pattern was similar with sheep [85], panda [86], bear [87] and Yak [88], suggesting that global glaciations and severely cold climates at this time had substantial evolutionary impact on the population size of terrestrial mammals [89].

As we known that musk deer is famous for secreting musk. Musk is a secreted external hormone or information compound that is stored in musk scent glands of the males of species within the family Moschidea [90]. Like those produced by muskrat (*Ondatra zibethicus L.*) and small Indian civet (*Viverricula indica*), the musk that musk glands of males secrete during the rutting season is not only an important pheromone for attracting females and mark territory, but also precious materials for pharmaceutical and perfume industries [91, 92]. Chemical analysis indicated that musk contains various ingredients, such as muscone, steroid compounds (cholestanol, cholesterol, and a number of the androstane derivatives), macrocyclic ketone, waxes, muscopyridine, and hydroxymuscopyridines, etc. [9, 10]. Fan et al. (2018) [93] has reported that testosterone and estradiol may play a major role in determining musk composition during the early stage of musk secretion but not during the course of musk maturation, which suggests that musk secretion may be promoted by increases in sex hormones in June. Other studies have shown that testosterone plays an important role in the seasonal development of musk glands [92], and oxytocin may regulate the function of muskrat scented glands by the locally expressed receptors [94]. Studies based on transcriptome [42, 43] and genetic analysis [35] have shown that a considerable number of genes involved in musk metabolism pathways, such as steroid biosynthesis, flavone and flavonol biosynthesis, terpenoid backbone biosynthesis, aldosterone-regulated sodium reabsorption was played a significant role in musk secretion. In this study, we identified 21 up-regulation DEGs which were closely associated with metabolism and response of steroid, terpenoid and hormone which were coincident with the previous reports [35, 42, 43]. Although there have been several studies on the secretion of musk, the genetic mechanisms of musk secretion are still poorly understood. Thus, further studies are needed to explore the musk secretion.

## Conclusion

Siberian musk deer once inhabited most of Asia, but today they are sharply declining and being endangered status due to overharvesting, natural disaster, and diseases. In this study, we report the first whole genome sequencing, assembly, and annotation of the wild Siberian musk deer. Comparative genomic analyses characterized genetic diversity, the population structure of Siberian musk deer, and even the genetic features associated with energy metabolism and adaptations in cold and high-altitude environments. The candidate genes identified in this study may be useful for understanding the mechanism of musk secretion. Collectively, the draft genome will provide a valuable resource for studying essential developmental processes in the musk deer, investigation evolution and providing the molecular breeding of this economically important species.

## Methods

### Sample collection, DNA and RNA isolation

Whole blood samples from two female and one male WSMD (DES, s190119001, s180119002, respectively) living at the Siberian musk deer breeding farm in Gachuurt village (45 km from Khan Khentii Strictly Protected Area), Khentii aimag, Mongolia, was collected during a routine veterinary examination. A mixture tissue sample (including liver, kidney, lung, heart, skin, and stomach) was collected from a female Siberian musk deer the naturally died. The genomic DNA was extracted from blood samples with Qiagen DNA blood and tissue kit (Qiagen, Velencia, USA), and the total RNA was isolated from mixture tissue using TRIzol reagent (Qiagen, Hilden, the Netherlands) following the manufacturer’s protocols.

All collected samples were approved by the Mongolian Musk Deer Breeding Center and completed with the help of staff.

### Genome sequencing and assembly

The whole genome shotgun strategy based on the Illumina HiSeq Xten platform was used to sequence the genome of one female WSMD (DES). In total, 19 paired-end libraries with insert sizes of 250 bp, 450 bp, 2 kb, 5 kb, and 10 kb were constructed and sequenced with the 2 × 150 bp mode. (Additional file 1: Table S1). For libraries with insert sizes > 1 kb, the DNA fragments were circularized by self-ligation. The raw reads were cleaned according to: 1) trimming adaptors; 2) filtering reads with N% > 0.1; 3) filtering reads with low-quality (score < 5) bases% > 0.2. Duplicated reads were also filtered. To check the quality of the libraries, the reads were mapped to an assembly of a close species (*Cervus elaphus*) with BWA mem (v0.7.12) [95] to re-estimate the insert sizes. The genomic sequence was assembled de novo by AllPaths-LG (v52488) [96]. Gaps were filled by short-fragment libraries with GapCloser (v1.12) -p 25 -l 150 in SOAPdenovo2 [97]. The consistency was evaluated by re-mapping the short-fragment libraries to the assembled genome with BWA mem (v0.7.12) [95] and then summarized with Picard (v2.3.0, https://broadinstitute.github.io/picard/) (Additional file 1: Table S3). The completeness was evaluated by BUSCO (v2.0) [53], based on 4104 universal single-copy orthologs in mammalian set (Additional file 1: Table S4). 262 nuclear sequences belonging to Moschus were fetched from Genbank and aligned to the assembled genome with exonerate (v2.2.0) [98] est2genome (Additional file 1: Table S5).

### Genome comparison of Siberian musk deer and forest musk deer

The genome of forest musk deer assembled by Fan et al. was downloaded from (http://gigadb.org/dataset/100411). We performed whole-genome alignment between the Siberian musk deer and forest musk deer assembly using mummer4 (nucmer -l 100 -c 500 --maxmatch) [56]. The alignment was filtered with minimum alignment length of 5 Kb (delta-filter -l 5000), and the difference was summarized using dnadiff.

### Genome annotation

Transposable elements (TEs) in the genome were identified by RepeatMasker (v4.0.6) -s -nolow (http://www.repeatmasker.org/) (Additional file 1: Table S6). TEs from both homology-based searchings against known ruminantia and mammalian sequences in Repbase (v16.10) [99], as well as de novo prediction by RepeatModeler (v1.0.8) were combined and masked. The TEs of other genomes for comparison were fetched from RepeatMasker datasets online (http://repeatmasker.org/genomicDatasets/RMGenomic Datasets.html) (Additional file 1: Table S7).

Protein-coding genes were annotated by three approaches (Additional file 1: Table S8). Firstly, AUGUSTUS (v3.0.1) [100], GENEID (v1.4.4) [101], GeneMark_ES (v2.3e) [102], GlimmerHMM (v3.0.2) [103] and SNAP (v2013-11-29) [104] were applied for ab initio scan of gene structures. Secondly, the longest protein sequences of each gene from humans, cattle, dogs, sheep, pig, mouse and goat were fetched from RefSeq and projected to the assembled genome. The rough alignment was performed by genBlastA (v1.0.1) [105], with protein coverage greater than 30%. Then precise alignment aware of gene structure on the target DNA sequences was performed by GeneWise (v2.4.1) [106]. Thirdly, RNA-Seq data of mixture tissues, as well as previously reported RNA-Seq data [42] of musk gland (SRR2098995, SRR2098996) and heart (SRR2142357) were mapped to the genome by Tophat2 (v2.1.1) [107] (Additional file 1: Table S9). The transcripts were assembled with Cufflinks (v2.2.1) [108] and merged with cuffmerge. Only transcripts with putative coding regions were preserved with TransDecoder (v3.0.1) [109]. Finally, the three gene sets were merged by EVM [110] with a weight combination (GeneWise > Cufflinks > ab initio). As evaluated by BUSCO (v2.0) [53], EVM genes with only ab initio evidence were removed (Additional file 1: Figure S1), and the remaining genes were complemented and updated with GeneWise [106] (Additional file 1: Table S4).

### Gene family construction

Gene families among the musk deer and other mammals were constructed with the TreeFam pipeline [111] (Additional file 1: Table S10), as described in detail by Li et al. [112]. Protein sequences were downloaded from RefSeq, and the longest one of each gene was chosen. All-to-all pairwise blastp were performed with -e 1e-10. Local alignments were joined by solar, and the alignment length should cover at least 1/3 on both proteins. A h-score was calculated for each protein pair (p1, p2) based on the blast score: h-score = score(p1, p2)/max (score(p1, p1), score(p2, p2)). Homologous proteins were then clustered with hcluster_sg -w 5 -s 0.33 and the opossum as an outgroup. Multiple alignments for each protein cluster were performed by clustalo (v1.2.0) [113], which was translated to CDS alignment by treebest backtrans. Guided by the common tree from NCBI Taxonomy, the phylogenetic tree for each cluster was constructed by treebest best. Orthologs were inferred from the cluster with treebest nj -t dm -v. Solar, hcluster_sg, and treebest were obtained from https://sourceforge.net/p/treesoft/code/ HEAD/tree/branches/lh3/.

### Genome evolution

Four-fold degeneration sites were extracted from the CDS alignment of single-copy orthologs. They were concatenated to reconstruct the species tree with the NJ method by MEGA (v7.0.18) [114]. The species tree was calibrated by MCMCtree in PAML (v4.9) [115], using the following divergence time from TimeTree [116] (2.5% lower and upper bounds): cattle-sheep (10–40 Mya), cattle-pig (40–80 Mya), cattle-horse (55–90 Mya) and cattle-human (65–150 Mya).

The evolution of gene family size was inferred by CAFE (v3.1) [117] based on the homologous clusters. For families with significant size variations (family-wide *p*-value < 0.01), the branches with significant expansion and contraction were selected (Viterbi *p*-value < 0.01).

Based on the CDS alignment of single-copy orthologs, positively selected genes (PSGs) in the musk deer were identified by codeml in PAML (v4.9) [116]. Poorly aligned regions were first filtered by Gblocks (0.91b) [118]. Taking the musk deer as the foreground and other species as the background, the branch-site model (model = 2, NSsite = 2) with dN/dS ≤ 1 (fix_omega = 1, omega = 1) and dN/dS > 1 (fix_omega = 0) were compared. The genes with significant dN/dS > 1 were identified by the likelihood ratio test (*p* < 0.05, chi-square test), and the positively selected sites (PSSs) were identified by the Bayes Empirical Bayes (BEB) analysis. To reduce the impact of defective gene annotation, genes with successive PSSs or PSSs located at the head or tail of the alignment (within 10 amino acids) were filtered. We conducted enrichment of the gene families and PSGs using KOBAS (v.3.0) [119]. Go terms and KEGG pathways with corrected *p*-values < 0.05 were identified as significantly enriched.

### Genomic diversity and demography inference

Genomes of two additional musk deer (s190119001 and s180119002) were re-sequenced with the standard Illumina HiSeq protocol (2 × 150 bp). The reads were cleaned with Trimmomatic (v0.36) [120] and mapped to the assembled genome with BWA mem (v0.7.12) [1] (Additional file 1: Table S3). Duplicates were marked with Picard (v2.3.0), and Indel re-alignment was performed with GATK (v3.5) [121]. Variant calling was first performed for each sample with HaplotypeCaller -stand_call_conf 30 in the GVCF mode, which was then combined for joint genotyping with GenotypeGVCFs. SNPs were selected and filtered with VariantFiltration ‘QD < 2.0 || FS > 60.0 || MQ < 40.0 || MQRankSum < − 12.5 || ReadPosRankSum < − 8.0’. Only biallelic SNPs were preserved in the following analysis (Additional file 1: Table S11). The demographic inference was performed with the PSMC model (v0.6.5) [59]. The consensus sequences for each individual were constructed with vcftools vcf-consensus (v0.1.12) [122] and transformed into the fastq format compatible with the PSMC input. Recommended parameters for the PSMC analysis were adopted, and the plot was scaled with -u 1.1e-08 -g 5 as estimated by Chen et al. [123].

### RNA-Seq analysis

The RNA sequencing of mixture tissues was performed with the standard Illumina HiSeq protocol (2 × 150 bp). The RNA-Seq data [42] of two musk glands (SRR2098995, SRR2098996) and one heart tissue (SRR2142357) were downloaded from SRA. The raw reads were cleaned with Trimmomatic (v0.36) [120] and mapped to the assembled genome with STAR (v020201) [54] (Additional file 1: Table S12), which showed a higher mapping efficiency than Tophat2 (v2.1.1) [107]. Guided by the gene annotations, the transcripts were re-assembled with Cufflinks (v2.2.1) [108] and then merged with cuffmerge. Reads that mapped to exons were counted by HTSeq (v0.6.0) [124] and then normalized by the R package DESeq (v1.28.0) [125]. Differential expression analysis was performed with DESeq based on the negative binomial distribution (FDR < 0.05), and clustering analysis was performed with the R package NMF (v0.20.6) [126] (Additional file 1: Figure S2). Go terms and KEGG pathways were also performed by KOBAS(V3.0) with *p*-values < 0.05 were identified as significantly enriched.

## Supplementary information

**Additional file 1: **Contains **Tables S1-S15** and **Figures S1-S4** with detailed results for the Figures presented in the main manuscript.

**Additional file 2: Table S16.** Go enrichment of DEGs in the *M. moschiferus* (*p* < 0.05).

**Additional file 3: Table S17.** (Comparison of the genome assembly of Siberian musk deer and forest musk deer).

## Data Availability

The dataset supporting the conclusions of this article is available on NCBI BioProject and can be accessed using the accession number PRJNA574937.

## Abbreviations

AKR: Aldo-keto reductase
BEB: Bayes Empirical Bayes
BP: Biological processes
CC: Cellular components
CYP: Cytochrome P450
DEGs: Differentially expressed genes
EVM: EvidenceModeler
LINES: Long interspersed elements
LTRs: Long terminal repeats
MF: Molecular functions
Mya: Million years ago
PSGs: Positively selected genes
PSMC: Pair-wise Sequentially Markovian Coalescent
PSSs: Positively selected sites
SDR: Short chain dehydrogenase
SINES: Short interspersed elements
SNP: Single-nucleotide polymorphism
TEs: Transposable elements
