# Supplementary material for: Whole-genome sequencing of wild Siberian musk deer (Moschus moschiferus) provides insights into its genetic features
Source: BMC Genomics. 2020 Jan 31;21:108. doi: 10.1186/s12864-020-6495-2 (PMC6995116; doi:10.1186/s12864-020-6495-2)
Supplement: Supplementary file 1 — Additional file 1: Contains Tables S1-S15 and Figures S1-S4 with detailed results for the Figures presented in the main manuscript. [file 12864_2020_6495_MOESM1_ESM.docx]

**Supplementary material**

**Supplementary Figures**


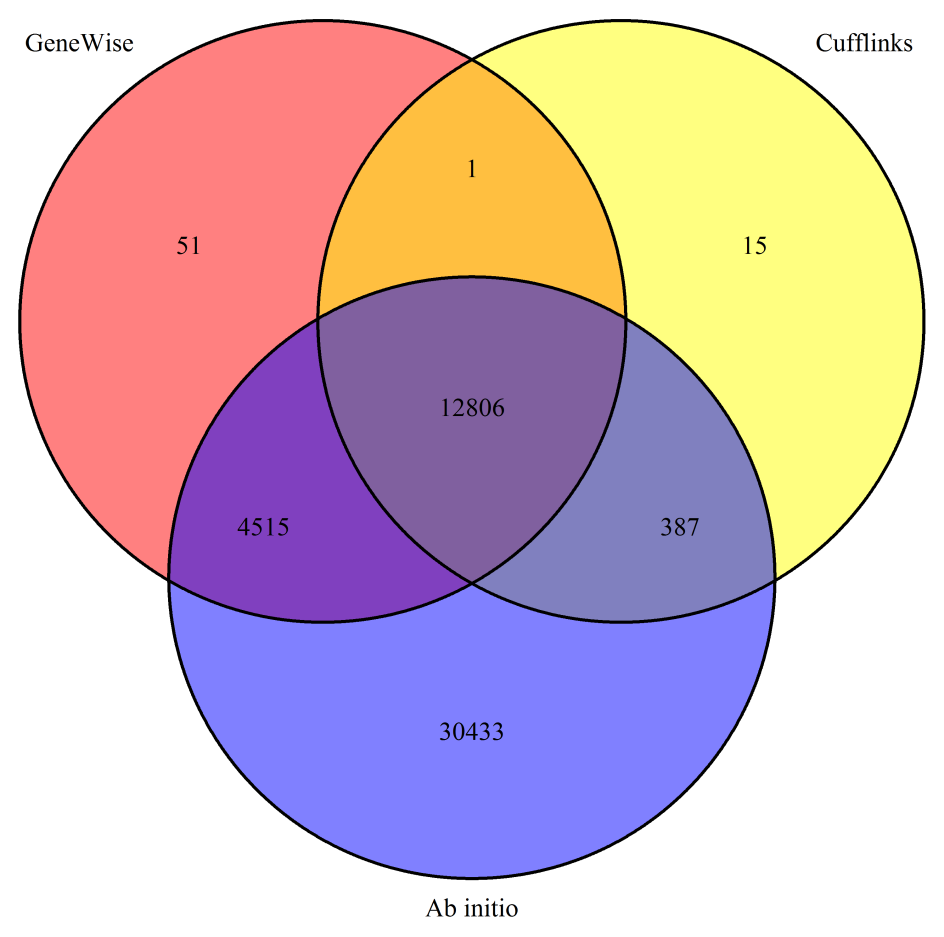


**Supplementary Figure S1. Summary of evidence for gene models predicted by EVM.** The gene numbers supported by ab initio methods, homology-based projection (GeneWise) and RNA-Seq assembly (Cufflinks) are shown. Genes with only ab initio supporting should be removed.


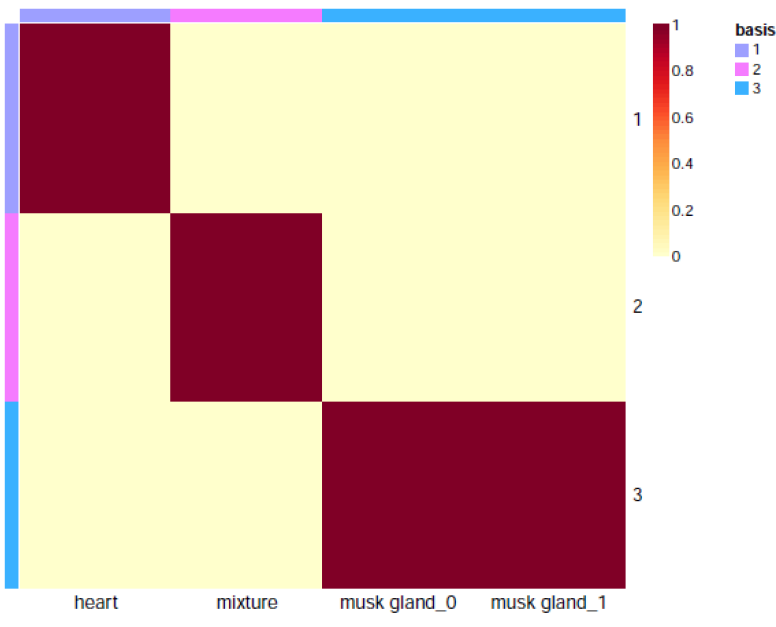


**Supplementary Figure S2. RNA-Seq sample clustering based on NMF.** The heatmap shows the mixture coefficient matrix with three bases.


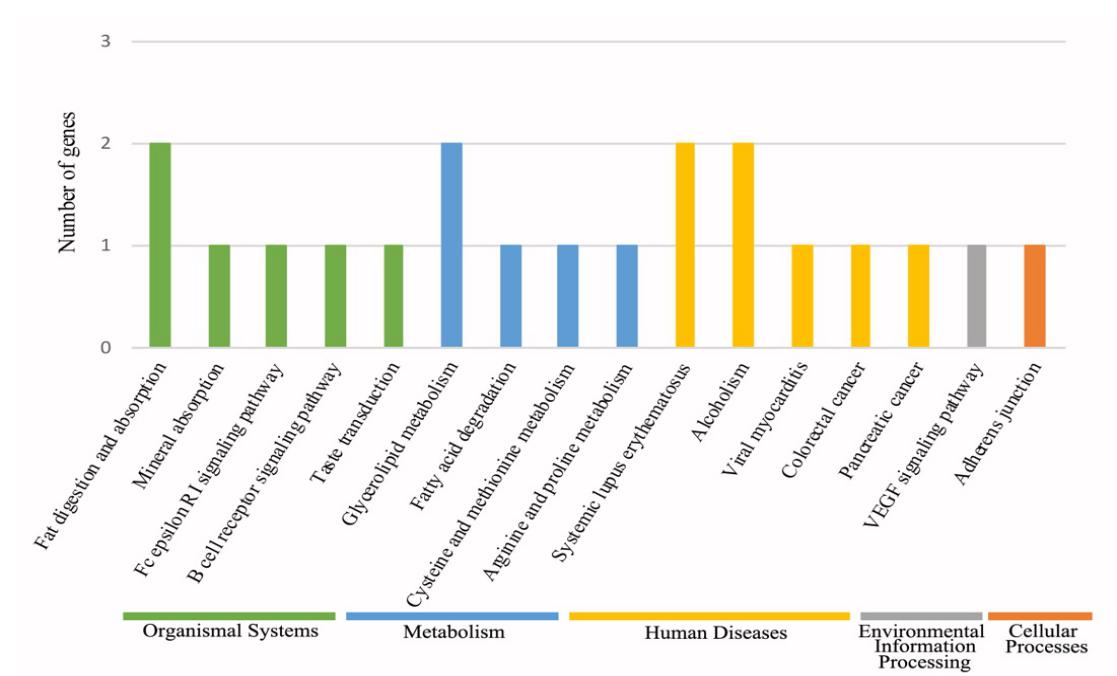


**Supplementary Figure S3. KEGG pathway enrichment of expended gene families in the Siberian musk deer.**


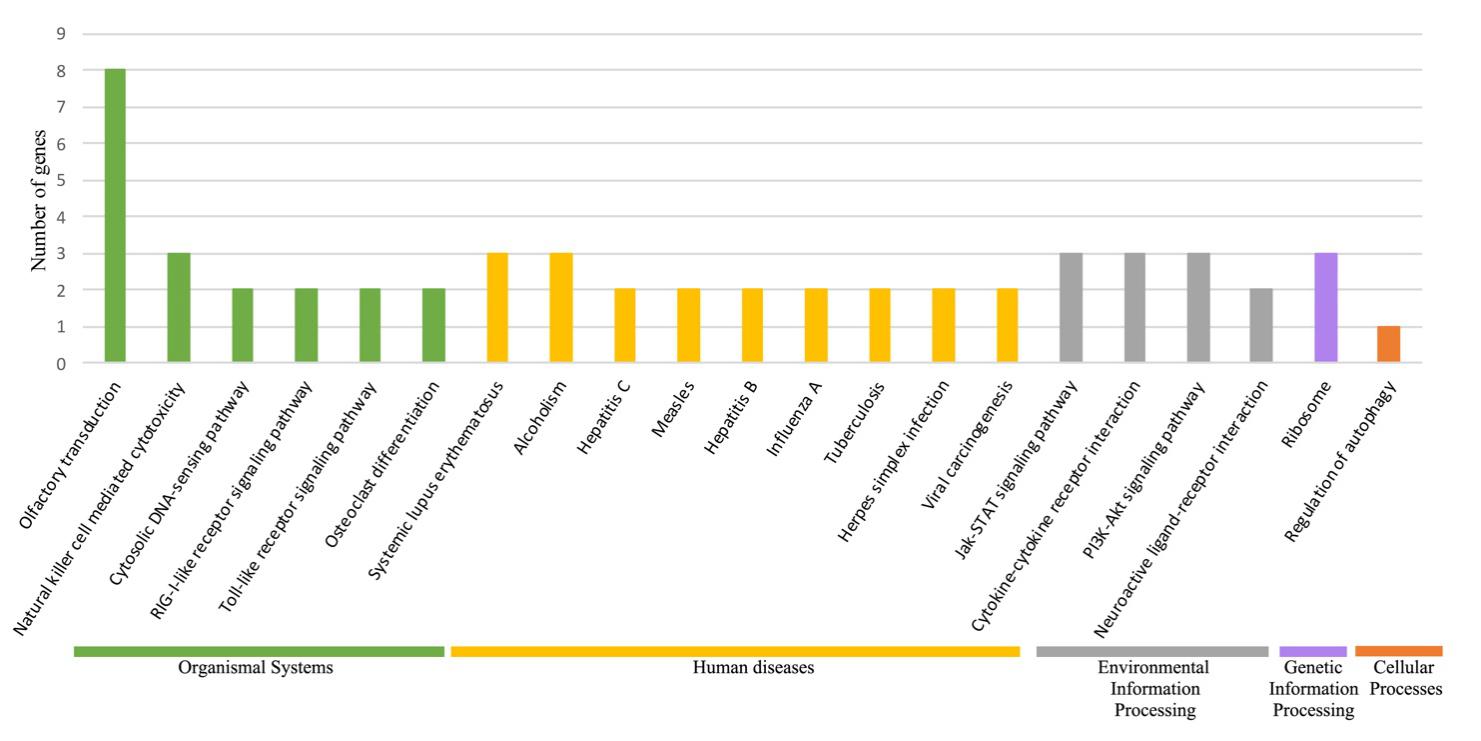


**Supplementary Figure S4. KEGG pathway enrichment of contracted gene families in the Siberian musk deer.**

**Supplementary Tables**

**Supplementary Table S1. Summary of genome library and sequencing data.**

| **Library** | **Library size (bp)** | **Lane** | **Raw base (Gb)** | **Clean base (Gb)** | **No duplication clean base (Gb)** | **Insert size (bp) ^a^** |
| --- | --- | --- | --- | --- | --- | --- |
| DES02299 | 250 | L4 | 19.56 | 19.55 | 17.67 | 213 |
| DES02299 | 250 | L5 | 23.35 | 23.32 | 21.01 | 221 |
| DES02300 | 250 | L4 | 24.21 | 24.17 | 21.75 | 209 |
| DES02300 | 250 | L5 | 22.61 | 22.56 | 20.24 | 218 |
| Sum of 250 bp library | | | 89.73 | 89.60 | 80.67 |  |
| DES02297 | 450 | L3 | 10.72 | 10.72 | 10.21 | 411 |
| DES02297 | 450 | L4 | 18.13 | 17.87 | 16.92 | 411 |
| DES02297 | 450 | L5 | 21.05 | 20.75 | 19.64 | 417 |
| DES02298 | 450 | L3 | 4.91 | 4.91 | 4.66 | 379 |
| DES02298 | 450 | L4 | 18.57 | 18.30 | 17.23 | 380 |
| DES02298 | 450 | L5 | 24.39 | 24.03 | 22.62 | 379 |
| Sum of 450 bp library | | | 97.77 | 96.58 | 91.28 |  |
| DEL01403 | 2000 | L1 | 22.22 | 21.61 | 19.08 | 1559 |
| DEL01403 | 2000 | L8 | 0.94 | 0.91 | 0.85 | 1719 |
| DEL01406 | 2000 | L1 | 21.86 | 21.50 | 18.82 | 2108 |
| DEL01406 | 2000 | L8 | 0.89 | 0.87 | 0.82 | 1758 |
| Sum of 2 Kb library | | | 45.91 | 44.90 | 39.58 |  |
| DEL01404 | 5000 | L1 | 22.97 | 22.64 | 17.47 | 4253 |
| DEL01407 | 5000 | L1 | 24.40 | 24.14 | 19.26 | 4091 |
| Sum of 5 Kb library | | | 47.36 | 46.78 | 36.73 |  |
| DEL01408 | 10000 | L1 | 14.95 | 13.23 | 11.15 | 5308 |
| DEL01408 | 10000 | L2 | 15.08 | 13.37 | 11.39 | 6826 |
| DEL01405 | 10000 | L1 | 15.82 | 14.74 | 12.42 | 8351 |
| Sum of 10 Kb library | | | 45.86 | 41.34 | 34.96 |  |
| Total | | | 326.64 | 319.20 | 283.22 |  |

^a^ Insert size was estimated by mapping the reads to a close assembled genome (*Cervus elaphus*) with BWA.

**Supplementary Table S2. Base content of the assembled genome.**

| **Base** | **Number (bp)** | **% of genome** |
| --- | --- | --- |
| A | 752,053,228 | 27.82 |
| T | 751,124,687 | 27.79 |
| C | 543,478,399 | 20.11 |
| G | 543,258,662 | 20.10 |
| N | 113,260,403 | 4.19 |
| Total | 2,703,175,379 | 100 |
| GC | 1,086,737,061 | 41.96 ^a^ |

^a^ GC content = (G + C) / (A + T + G + C).

**Supplementary Table S3. Genome mapping statistics for three individual musk deer by BWA.**

| **Statistics** | **ref (short library)** | **s180119001** | **s180119002** |
| --- | --- | --- | --- |
| Mapping rate of read pairs | 98.00% | 97.86% | 97.99% |
| Properly paired rate | 93.16% | 91.46% | 91.85% |
| Mean coverage ^a^ | 39.04X | 8.59X | 9.07X |
| Genome coverage ≥1X | 97.21% | 96.36% | 96.49% |
| Genome coverage ≥5X | 96.64% | 86.14% | 89.06% |
| Genome coverage ≥10X | 96.06% | 38.04% | 43.06% |
| Genome coverage ≥15X | 95.52% | 5.33% | 6.80% |
| Genome coverage ≥20X | 94.85% | 0.67% | 0.80% |
| Genome coverage ≥30X | 87.98% | 0.26% | 0.27% |

^a^ After all default filters are applied by Picard.

**Supplementary Table S4. Assessment of the genome assembly and predicted gene sets by 4,104 mammalian BUSCOs.**

| **Genome / gene set** | **Total predicted genes** | **Complete BUSCOs (C = S + D)** | **Complete and single-copy BUSCOs (S)** | **Complete and duplicated BUSCOs (D)** | **Fragmented BUSCOs (F)** | **Missing BUSCOs (M)** |
| --- | --- | --- | --- | --- | --- | --- |
| Genome assembly | - | 93.30% | 91.30% | 2.00% | 3.30% | 3.40% |
| EVM (all predictions) | 48208 | 85.00% | 82.60% | 2.40% | 10.80% | 4.20% |
| EVM (excluding only ab initio supporting) | 17775 | 83.80% | 82.80% | 1.00% | 10.60% | 5.60% |
| GeneWise (best projection per gene) | 14897 | 92.20% | 91.40% | 0.80% | 0.70% | 7.10% |
| GeneWise (all projections per gene) | 21507 | 92.50% | 89.50% | 3.00% | 0.90% | 6.60% |
| GeneWise (best projection per gene) supplemented with EVM (excluding only ab initio supporting) | 19363 | 97.00% | 94.80% | 2.20% | 2.10% | 0.90% |

**Supplementary Table S5. Alignment of the genome assembly and Moschus nucleotide sequences from Genbank.**

| **Sequence type** | **Number of sequences with alignment length/nucleotide length** | | |
| --- | --- | --- | --- |
|  | **>95%** | **>80%** | **0%** |
| mRNA | 168 | 0 | 7 |
| ncRNA | 5 | 0 | 1 |
| MHC | 33 | 0 | 12 |
| microsatellite | 6 | 2 | 26 |
| chromosome Y | 0 | 0 | 2 |

**Supplementary Table S6. Proportion of genome masked by transposable elements with different methods.**

| **Class** | **Sub-class** | **Ruminantia (Repbase)** | **Mammal (Repbase)** | **De novo (RepeatModeler)** | **Combined** |
| --- | --- | --- | --- | --- | --- |
| LINE |  | 26.55% | 25.24% | 30.03% | 30.37% |
|  | CR1 | 0.25% | 0.26% | 0.00% | 0.11% |
|  | L1 | 11.86% | 10.66% | 12.25% | 13.89% |
|  | L2 | 2.27% | 2.23% | 0.46% | 1.14% |
|  | RTE-BovB | 12.10% | 12.05% | 16.65% | 15.00% |
|  | RTE-X | 0.07% | 0.04% | 0.00% | 0.06% |
|  | Other | 0.00% | 0.00% | 0.67% | 0.17% |
| SINE |  | 11.35% | 11.37% | 1.30% | 4.78% |
|  | 5S-Deu-L2 | 0.01% | 0.01% | 0.00% | 0.00% |
|  | Core-RTE | 1.98% | 1.93% | 0.00% | 0.48% |
|  | L2 | 0.00% | 0.01% | 0.00% | 0.00% |
|  | MIR | 2.04% | 2.32% | 0.75% | 1.19% |
|  | tRNA | 1.53% | 1.54% | 0.55% | 1.50% |
|  | tRNA-Core-RTE | 5.78% | 5.55% | 0.00% | 1.61% |
|  | tRNA-RTE | 0.01% | 0.01% | 0.00% | 0.00% |
| LTR |  | 4.23% | 4.70% | 2.82% | 4.42% |
|  | ERV1 | 1.24% | 1.57% | 1.22% | 1.62% |
|  | ERVK | 0.31% | 0.40% | 0.12% | 0.14% |
|  | ERVL | 1.06% | 1.10% | 0.53% | 1.06% |
|  | ERVL-MaLR | 1.45% | 1.47% | 0.91% | 1.42% |
|  | Gypsy | 0.09% | 0.08% | 0.00% | 0.08% |
|  | Other | 0.08% | 0.08% | 0.04% | 0.10% |
| DNA |  | 2.11% | 2.33% | 1.30% | 2.27% |
|  | CMC-EnSpm | 0.00% | 0.00% | 0.03% | 0.02% |
|  | PiggyBac | 0.01% | 0.01% | 0.00% | 0.01% |
|  | TcMar | 0.01% | 0.01% | 0.00% | 0.01% |
|  | TcMar-Mariner | 0.05% | 0.12% | 0.12% | 0.14% |
|  | TcMar-Tc2 | 0.04% | 0.04% | 0.00% | 0.04% |
|  | TcMar-Tigger | 0.43% | 0.51% | 0.34% | 0.53% |
|  | hAT | 0.04% | 0.04% | 0.02% | 0.04% |
|  | hAT-Ac | 0.02% | 0.02% | 0.00% | 0.01% |
|  | hAT-Blackjack | 0.09% | 0.09% | 0.05% | 0.09% |
|  | hAT-Charlie | 1.12% | 1.18% | 0.63% | 1.08% |
|  | hAT-Tag1 | 0.01% | 0.01% | 0.00% | 0.01% |
|  | hAT-Tip100 | 0.25% | 0.25% | 0.05% | 0.21% |
|  | Other | 0.04% | 0.05% | 0.06% | 0.08% |
| RC | Helitron | 0.01% | 0.01% | 0.01% | 0.02% |
| Other |  | 0.02% | 0.02% | 3.14% | 2.58% |
| Total interspersed | | 44.27% | 43.67% | 38.60% | 44.44% |

**Supplementary Table S7. Comparison of transposable elements with other mammalian genomes.**

| **Class** | **Sub-Class** | **Musk deer** | **Cattle** | **Pig** | **Human** | **Mouse** |
| --- | --- | --- | --- | --- | --- | --- |
| LINE |  | 30.37% | 27.14% | 19.09% | 20.73% | 19.85% |
|  | CR1 | 0.11% | 0.29% | 0.34% | 0.39% | 0.07% |
|  | L1 | 13.89% | 12.45% | 15.72% | 16.62% | 19.34% |
|  | L2 | 1.14% | 2.50% | 2.90% | 3.56% | 0.42% |
|  | RTE-BovB | 15.00% | 11.82% | 0.04% | 0.04% | 0.01% |
|  | RTE-X | 0.06% | 0.08% | 0.09% | 0.12% | 0.01% |
|  | Other | 0.17% | 0.00% | 0.00% | 0.00% | 0.00% |
| SINE |  | 4.78% | 11.14% | 12.73% | 12.79% | 7.46% |
|  | 5S-Deu-L2 | 0.00% | 0.01% | 0.01% | 0.01% | 0.00% |
|  | Alu | 0.00% | 0.00% | 0.00% | 10.03% | 2.43% |
|  | B2 | 0.00% | 0.00% | 0.00% | 0.00% | 2.19% |
|  | B4 | 0.00% | 0.00% | 0.00% | 0.00% | 2.14% |
|  | Core-RTE | 0.48% | 2.17% | 0.00% | 0.00% | 0.00% |
|  | ID | 0.00% | 0.00% | 0.00% | 0.00% | 0.16% |
|  | MIR | 1.19% | 2.16% | 2.46% | 2.72% | 0.54% |
|  | tRNA | 1.50% | 1.49% | 10.24% | 0.01% | 0.00% |
|  | tRNA-Core-RTE | 1.61% | 5.29% | 0.00% | 0.00% | 0.00% |
|  | tRNA-RTE | 0.00% | 0.02% | 0.02% | 0.02% | 0.00% |
| LTR |  | 4.42% | 4.70% | 4.41% | 8.84% | 11.70% |
|  | ERV1 | 1.62% | 1.36% | 1.17% | 2.77% | 1.14% |
|  | ERVK | 0.14% | 0.64% | 0.09% | 0.30% | 4.81% |
|  | ERVL | 1.06% | 1.05% | 1.24% | 1.89% | 1.19% |
|  | ERVL-MaLR | 1.42% | 1.48% | 1.71% | 3.60% | 4.52% |
|  | Gypsy | 0.08% | 0.10% | 0.12% | 0.17% | 0.02% |
|  | Other | 0.10% | 0.07% | 0.08% | 0.11% | 0.02% |
| DNA |  | 2.27% | 2.13% | 2.36% | 3.51% | 1.09% |
|  | CMC-EnSpm | 0.02% | 0.00% | 0.00% | 0.00% | 0.00% |
|  | MULE-MuDR | 0.00% | 0.00% | 0.00% | 0.02% | 0.00% |
|  | PiggyBac | 0.01% | 0.01% | 0.01% | 0.02% | 0.00% |
|  | TcMar | 0.01% | 0.01% | 0.01% | 0.01% | 0.00% |
|  | TcMar-Mariner | 0.14% | 0.05% | 0.02% | 0.09% | 0.01% |
|  | TcMar-Tc2 | 0.04% | 0.04% | 0.05% | 0.05% | 0.01% |
|  | TcMar-Tigger | 0.53% | 0.44% | 0.50% | 1.22% | 0.20% |
|  | hAT | 0.04% | 0.04% | 0.05% | 0.05% | 0.02% |
|  | hAT-Ac | 0.01% | 0.02% | 0.02% | 0.02% | 0.02% |
|  | hAT-Blackjack | 0.09% | 0.09% | 0.10% | 0.11% | 0.03% |
|  | hAT-Charlie | 1.08% | 1.12% | 1.26% | 1.52% | 0.69% |
|  | hAT-Tag1 | 0.01% | 0.01% | 0.01% | 0.02% | 0.01% |
|  | hAT-Tip100 | 0.21% | 0.26% | 0.29% | 0.34% | 0.08% |
|  | Other | 0.08% | 0.04% | 0.04% | 0.04% | 0.02% |
| RC | Helitron | 0.02% | 0.01% | 0.01% | 0.01% | 0.00% |
| Other |  | 2.58% | 0.03% | 0.03% | 0.17% | 0.40% |
| Total interspersed | | 44.44% | 45.14% | 38.66% | 46.07% | 40.53% |

**Supplementary Table S8. Statistics of protein-coding genes predicted by different methods.**

| **Method** | | **Gene number** | **Average gene length (bp)** | **Average CDS length (bp)** | **Average exon per gene** | **Average exon length (bp)** | **Average intron length (bp)** |
| --- | --- | --- | --- | --- | --- | --- | --- |
| Ab initio prediction | AUGUSTUS | 22373 | 48549.50 | 1488.34 | 8.81 | 168.94 | 6025.66 |
|  | GENEID | 43259 | 28364.71 | 878.36 | 5.20 | 168.90 | 6531.69 |
|  | GlimmerHMM | 115672 | 9418.30 | 628.84 | 2.84 | 221.29 | 4772.36 |
|  | SNAP | 128763 | 12583.31 | 763.72 | 4.90 | 155.99 | 3033.93 |
|  | GeneMark-ES/ET | 80034 | 6467.94 | 1347.62 | 10.81 | 124.64 | 521.85 |
| Homology-based projection | H.sapiens | 20917 | 27786.58 | 1530.49 | 7.82 | 195.69 | 3849.27 |
|  | B.taurus | 22475 | 26188.20 | 1469.84 | 7.62 | 192.96 | 3735.46 |
|  | C.familiaris | 20352 | 27427.81 | 1525.89 | 7.91 | 193.02 | 3750.93 |
|  | O.aries | 21165 | 26842.68 | 1480.25 | 7.77 | 190.47 | 3745.36 |
|  | S.scrofa | 24533 | 22165.29 | 1315.99 | 6.78 | 194.03 | 3605.67 |
|  | M.musculus | 22707 | 25193.13 | 1477.05 | 7.21 | 204.94 | 3820.68 |
|  | C.hircus | 21350 | 27982.57 | 1541.66 | 8.04 | 191.78 | 3756.41 |
| RNA-Seq assembly |  | 22484 | 32990.35 | 2740.55 | 7.44 | 368.55 | 4686.33 |

**Supplementary Table S9. Mapping RNA-Seq data to the genome assembly using Tophat2.**

| **Number of read pairs** | **musk gland (SRR2098996)** | **musk gland (SRR2098995)** | **heart (SRR2142357)** | **mixture** |
| --- | --- | --- | --- | --- |
| Clean input | 22181940 | 22151491 | 23756035 | 17323786 |
| Aligned | 16550015 | 16805670 | 7726419 | 11947542 |
| Multiple alignment | 1756761 | 1985976 | 733740 | 1286051 |
| Discordant alignment | 801313 | 843440 | 251662 | 416235 |

**Supplementary Table S10. Statistics of mammalian genes for ortholog construction.**

| **Species** | **Gene number ^a^** | **Homolog cluster ^b^** | **Ortholog ^c^** |
| --- | --- | --- | --- |
| M. Moschiferus | 19363 | 15959 | 17336 |
| B. taurus | 21435 | 16508 | 18279 |
| O. aries | 20534 | 16348 | 18025 |
| C. hircus | 20593 | 16406 | 18108 |
| O. virginianus | 21059 | 16521 | 18381 |
| S. scrofa | 24105 | 16038 | 17682 |
| E. caballus | 20264 | 16136 | 17862 |
| C. familiaris | 19849 | 16215 | 17770 |
| H. sapiens | 20366 | 16297 | 17719 |
| M. musculus | 22459 | 16302 | 17682 |
| M. domestica | 20891 | 17153 | 16253 |

^a^ Genes in NCBI RefSeq.

^b^ Cluster based on all-to-all blast.

^c^ Inferred by treebest.

**Supplementary Table S11. Number of SNPs identified in the three genomes.**

| Sample | SNP count | Heterozygous SNP count | Missing count | Private count | Ts/tv ratio |
| --- | --- | --- | --- | --- | --- |
| DES raw | 3791194 | 2957540 | 33169 | 857168 | 1.07 |
| s180119001 raw | 3998119 | 2252262 | 195739 | 640959 | 1.3 |
| s180119002 raw | 4247365 | 2479409 | 178324 | 841207 | 1.33 |
| Total raw | 6157622 |  |  |  | 1.3 |
| DES preserved | 2619369 | 2420974 | 1918 | 533900 | 1.74 |
| s180119001 preserved | 3135466 | 2002344 | 67482 | 575059 | 1.84 |
| s180119002 preserved | 3378692 | 2227725 | 57424 | 781376 | 1.85 |
| Total preserved | 4817590 |  |  |  | 1.84 |

**Supplementary Table S12. Statistics of RNA-Seq data by STAR mapping and gene quantification.**

| **Analysis** | **Statistics** | **musk gland** | **musk gland** | **heart** | **mixture** |
| --- | --- | --- | --- | --- | --- |
| STAR mapping | Number of input reads | 22181940 | 22151491 | 23756035 | 17323786 |
|  | % of unique-mapping reads | 85.14% | 85.14% | 62.52% | 79.41% |
|  | % of multi-mapping reads | 9.32% | 10.60% | 13.47% | 7.58% |
|  | % of unmapped reads | 5.53% | 4.26% | 24.00% | 13.01% |
|  | % of chimeric reads | 0.00% | 0.00% | 0.00% | 0.00% |
| Cufflinks assembly | Number of genes | 28051 | 27730 | 25081 | 44271 |
|  | Number of isoforms | 49879 | 49563 | 43528 | 61196 |
| HTSeq counting | Number of reads assigned to unique gene | 15203536 | 15314382 | 9823624 | 9716870 |
|  | Number of reads not assigned to any gene | 1081006 | 1198336 | 765275 | 998417 |
|  | Number of reads assigned to more than one gene | 2601886 | 2347012 | 4263690 | 3041129 |
|  | Number of reads with more than one alignment | 5816676 | 6659706 | 7249749 | 3112951 |

**Supplementary Table S13. GO enrichment analysis of expended gene families in the Siberian musk deer.**

| **Go ID** | **GO Term** | **GO class** | **Count** | ***p*-value** | **Corrected *p*-value** |
| --- | --- | --- | --- | --- | --- |
| GO:0003846 | 2-acylglycerol O-acyltransferase activity | MF | 2 | 9.74E-06 | 0.00689573 |
| GO:0016020 | membrane | CC | 15 | 2.48E-05 | 0.00689573 |
| GO:0031982 | vesicle | CC | 10 | 2.52E-05 | 0.00689573 |
| GO:0044421 | extracellular region part | CC | 10 | 3.12E-05 | 0.00689573 |
| GO:0006071 | glycerol metabolic process | BP | 2 | 5.93E-05 | 0.01049012 |
| GO:0019400 | alditol metabolic process | BP | 2 | 8.00E-05 | 0.01107394 |
| GO:0031988 | membrane-bounded vesicle | CC | 9 | 0.00012787 | 0.01107394 |
| GO:0005576 | extracellular region | CC | 10 | 0.00013052 | 0.01107394 |
| GO:0070062 | extracellular exosome | CC | 8 | 0.00013755 | 0.01107394 |
| GO:1903561 | extracellular vesicle | CC | 8 | 0.00014216 | 0.01107394 |
| GO:0043230 | extracellular organelle | CC | 8 | 0.00014252 | 0.01107394 |
| GO:0016411 | acylglycerol O-acyltransferase activity | MF | 2 | 0.00015016 | 0.01107394 |
| GO:0006335 | DNA replication-dependent nucleosome assembly | BP | 2 | 0.00019336 | 0.01222331 |
| GO:0034723 | DNA replication-dependent nucleosome organization | BP | 2 | 0.00019336 | 0.01222331 |
| GO:0051290 | protein heterotetramerization | BP | 2 | 0.00026825 | 0.01559294 |
| GO:0000183 | chromatin silencing at rDNA | BP | 2 | 0.00028191 | 0.01559294 |
| GO:0019432 | triglyceride biosynthetic process | BP | 2 | 0.00035517 | 0.01801647 |
| GO:0046463 | acylglycerol biosynthetic process | BP | 2 | 0.00038679 | 0.01801647 |
| GO:0046460 | neutral lipid biosynthetic process | BP | 2 | 0.00038679 | 0.01801647 |
| GO:0008374 | O-acyltransferase activity | MF | 2 | 0.00041975 | 0.01857379 |
| GO:0005884 | actin filament | CC | 2 | 0.00096725 | 0.04076272 |
| GO:0045815 | positive regulation of gene expression, epigenetic | BP | 2 | 0.00107088 | 0.04307872 |

**Supplementary Table S14. GO enrichment analysis of contracted gene families in the Siberian musk deer.**

| **Go ID** | **GO Term** | **GO class** | **Count** | **p-value** | **Corrected *p*-value** |
| --- | --- | --- | --- | --- | --- |
| GO:0009987 | cellular process | CC | 40 | 3.00E-12 | 4.22E-09 |
| GO:0050794 | regulation of cellular process | BP | 33 | 4.56E-11 | 3.21E-08 |
| GO:0050789 | regulation of biological process | BP | 33 | 1.91E-10 | 8.93E-08 |
| GO:0044464 | cell part | CC | 39 | 3.96E-10 | 1.18E-07 |
| GO:0005623 | cell | CC | 39 | 4.19E-10 | 1.18E-07 |
| GO:0004888 | transmembrane signaling receptor activity | MF | 13 | 8.59E-10 | 1.85E-07 |
| GO:0065007 | biological regulation | BP | 33 | 9.21E-10 | 1.85E-07 |
| GO:0050907 | detection of chemical stimulus involved in sensory perception | BP | 9 | 1.31E-09 | 2.28E-07 |
| GO:0099600 | transmembrane receptor activity | MF | 13 | 1.46E-09 | 2.28E-07 |
| GO:0038023 | signaling receptor activity | MF | 13 | 2.33E-09 | 3.27E-07 |
| GO:0009593 | detection of chemical stimulus | BP | 9 | 2.87E-09 | 3.67E-07 |
| GO:0050906 | detection of stimulus involved in sensory perception | BP | 9 | 3.51E-09 | 4.01E-07 |
| GO:0007606 | sensory perception of chemical stimulus | BP | 9 | 3.71E-09 | 4.01E-07 |
| GO:0004984 | olfactory receptor activity | MF | 8 | 1.27E-08 | 1.19E-06 |
| GO:0050911 | detection of chemical stimulus involved in sensory perception of smell | BP | 8 | 1.27E-08 | 1.19E-06 |
| GO:0050896 | response to stimulus | BP | 27 | 1.42E-08 | 1.25E-06 |
| GO:0004872 | receptor activity | MF | 13 | 1.89E-08 | 1.48E-06 |
| GO:0060089 | molecular transducer activity | MF | 13 | 1.89E-08 | 1.48E-06 |
| GO:0004930 | G-protein coupled receptor activity | MF | 10 | 2.03E-08 | 1.50E-06 |
| GO:0007608 | sensory perception of smell | BP | 8 | 2.23E-08 | 1.57E-06 |
| GO:0044700 | single organism signaling | BP | 23 | 2.78E-08 | 1.81E-06 |
| GO:0023052 | signaling | BP | 23 | 2.84E-08 | 1.81E-06 |
| GO:0007154 | cell communication | BP | 23 | 3.04E-08 | 1.86E-06 |
| GO:0042221 | response to chemical | BP | 19 | 3.41E-08 | 1.93E-06 |
| GO:0004871 | signal transducer activity | MF | 13 | 3.44E-08 | 1.93E-06 |
| GO:0007165 | signal transduction | BP | 22 | 3.95E-08 | 2.13E-06 |
| GO:0051606 | detection of stimulus | BP | 9 | 4.46E-08 | 2.32E-06 |
| GO:0007186 | G-protein coupled receptor signaling pathway | BP | 11 | 1.03E-07 | 5.15E-06 |
| GO:0005886 | plasma membrane | CC | 19 | 6.97E-07 | 3.38E-05 |
| GO:0007600 | sensory perception | BP | 9 | 9.01E-07 | 4.22E-05 |
| GO:0071944 | cell periphery | CC | 19 | 9.64E-07 | 4.37E-05 |
| GO:0051716 | cellular response to stimulus | BP | 22 | 1.30E-06 | 5.72E-05 |
| GO:0016021 | integral component of membrane | CC | 19 | 2.17E-06 | 9.24E-05 |
| GO:0044271 | cellular nitrogen compound biosynthetic process | BP | 18 | 2.86E-06 | 1.18E-04 |
| GO:0031224 | intrinsic component of membrane | CC | 19 | 3.07E-06 | 1.23E-04 |
| GO:0032501 | multicellular organismal process | BP | 21 | 6.31E-06 | 2.46E-04 |
| GO:0031323 | regulation of cellular metabolic process | BP | 19 | 7.50E-06 | 2.85E-04 |
| GO:0010467 | gene expression | BP | 18 | 8.63E-06 | 3.19E-04 |
| GO:0031326 | regulation of cellular biosynthetic process | BP | 16 | 9.39E-06 | 3.39E-04 |
| GO:0050877 | neurological system process | BP | 9 | 1.02E-05 | 3.58E-04 |
| GO:0009889 | regulation of biosynthetic process | BP | 16 | 1.11E-05 | 3.81E-04 |
| GO:0051171 | regulation of nitrogen compound metabolic process | BP | 16 | 1.30E-05 | 4.34E-04 |
| GO:0034645 | cellular macromolecule biosynthetic process | BP | 17 | 1.41E-05 | 4.62E-04 |
| GO:0003677 | DNA binding | MF | 12 | 1.51E-05 | 4.83E-04 |
| GO:0019222 | regulation of metabolic process | BP | 19 | 1.61E-05 | 5.03E-04 |
| GO:0000788 | nuclear nucleosome | CC | 3 | 1.73E-05 | 5.27E-04 |
| GO:2000112 | regulation of cellular macromolecule biosynthetic process | BP | 15 | 1.80E-05 | 5.38E-04 |
| GO:0009059 | macromolecule biosynthetic process | BP | 17 | 2.18E-05 | 6.39E-04 |
| GO:0010556 | regulation of macromolecule biosynthetic process | BP | 15 | 2.53E-05 | 7.26E-04 |
| GO:0080090 | regulation of primary metabolic process | BP | 18 | 2.73E-05 | 7.67E-04 |
| GO:0060255 | regulation of macromolecule metabolic process | BP | 18 | 2.79E-05 | 7.68E-04 |
| GO:0044260 | cellular macromolecule metabolic process | BP | 22 | 2.88E-05 | 7.78E-04 |
| GO:0016020 | membrane | CC | 23 | 3.13E-05 | 8.31E-04 |
| GO:0002725 | negative regulation of T cell cytokine production | BP | 2 | 4.68E-05 | 1.20E-03 |
| GO:0044425 | membrane part | CC | 19 | 4.68E-05 | 1.20E-03 |
| GO:0010468 | regulation of gene expression | BP | 15 | 4.93E-05 | 1.23E-03 |
| GO:0044249 | cellular biosynthetic process | BP | 18 | 5.00E-05 | 1.23E-03 |
| GO:0044699 | single-organism process | BP | 28 | 5.39E-05 | 1.31E-03 |
| GO:0009058 | biosynthetic process | BP | 18 | 7.84E-05 | 1.87E-03 |
| GO:0044763 | single-organism cellular process | BP | 26 | 8.44E-05 | 1.98E-03 |
| GO:0003676 | nucleic acid binding | MF | 14 | 8.65E-05 | 1.99E-03 |
| GO:0043170 | macromolecule metabolic process | BP | 22 | 9.65E-05 | 2.19E-03 |
| GO:0016070 | RNA metabolic process | BP | 15 | 0.00010172 | 2.27E-03 |
| GO:0030101 | natural killer cell activation | BP | 3 | 0.00010339 | 2.27E-03 |
| GO:0019219 | regulation of nucleobase-containing compound metabolic process | BP | 14 | 0.00010743 | 2.32E-03 |
| GO:0034641 | cellular nitrogen compound metabolic process | BP | 18 | 0.00011343 | 2.42E-03 |
| GO:0006355 | regulation of transcription, DNA-templated | BP | 13 | 0.00011737 | 2.46E-03 |
| GO:1903506 | regulation of nucleic acid-templated transcription | BP | 13 | 0.00012399 | 2.56E-03 |
| GO:2001141 | regulation of RNA biosynthetic process | BP | 13 | 0.00013055 | 2.66E-03 |
| GO:0051252 | regulation of RNA metabolic process | BP | 13 | 0.0001776 | 3.57E-03 |
| GO:0006351 | transcription, DNA-templated | BP | 13 | 0.00018303 | 3.62E-03 |
| GO:0097659 | nucleic acid-templated transcription | BP | 13 | 0.00019014 | 3.71E-03 |
| GO:0044237 | cellular metabolic process | BP | 23 | 0.00019654 | 3.79E-03 |
| GO:0034654 | nucleobase-containing compound biosynthetic process | BP | 14 | 0.00020514 | 3.90E-03 |
| GO:0005132 | type I interferon receptor binding | MF | 2 | 0.00022062 | 4.07E-03 |
| GO:0002710 | negative regulation of T cell mediated immunity | BP | 2 | 0.00022062 | 4.07E-03 |
| GO:0008152 | metabolic process | BP | 24 | 0.00022303 | 4.07E-03 |
| GO:1901576 | organic substance biosynthetic process | BP | 17 | 0.00022842 | 4.12E-03 |
| GO:0018130 | heterocycle biosynthetic process | BP | 14 | 0.00023387 | 4.14E-03 |
| GO:0000786 | nucleosome | CC | 3 | 0.00023555 | 4.14E-03 |
| GO:0019438 | aromatic compound biosynthetic process | BP | 14 | 0.00024143 | 4.19E-03 |
| GO:0032774 | RNA biosynthetic process | BP | 13 | 0.00026232 | 4.50E-03 |
| GO:0006807 | nitrogen compound metabolic process | BP | 18 | 0.00026655 | 4.52E-03 |
| GO:0044815 | DNA packaging complex | CC | 3 | 0.00027826 | 4.66E-03 |
| GO:0022626 | cytosolic ribosome | CC | 3 | 0.00030137 | 4.96E-03 |
| GO:1901362 | organic cyclic compound biosynthetic process | BP | 14 | 0.00030778 | 4.96E-03 |
| GO:0005488 | binding | MF | 28 | 0.00030885 | 4.96E-03 |
| GO:0003008 | system process | BP | 9 | 0.00031074 | 4.96E-03 |
| GO:0090304 | nucleic acid metabolic process | BP | 15 | 0.0003238 | 4.97E-03 |
| GO:0033139 | regulation of peptidyl-serine phosphorylation of STAT protein | BP | 2 | 0.00032545 | 4.97E-03 |
| GO:0033141 | positive regulation of peptidyl-serine phosphorylation of STAT protein | BP | 2 | 0.00032545 | 4.97E-03 |
| GO:0002724 | regulation of T cell cytokine production | BP | 2 | 0.00032545 | 4.97E-03 |
| GO:0002706 | regulation of lymphocyte mediated immunity | BP | 3 | 0.00035122 | 5.31E-03 |
| GO:0002719 | negative regulation of cytokine production involved in immune response | BP | 2 | 0.00035478 | 5.31E-03 |
| GO:0042501 | serine phosphorylation of STAT protein | BP | 2 | 0.00048446 | 7.17E-03 |
| GO:0098609 | cell-cell adhesion | BP | 6 | 0.00049983 | 7.32E-03 |
| GO:0022408 | negative regulation of cell-cell adhesion | BP | 3 | 0.0005093 | 7.38E-03 |
| GO:0002369 | T cell cytokine production | BP | 2 | 0.00051996 | 7.46E-03 |
| GO:0044238 | primary metabolic process | BP | 22 | 0.00057417 | 8.15E-03 |
| GO:0002701 | negative regulation of production of molecular mediator of immune response | BP | 2 | 0.00059465 | 8.28E-03 |
| GO:0002323 | natural killer cell activation involved in immune response | BP | 2 | 0.00059465 | 8.28E-03 |
| GO:0006334 | nucleosome assembly | BP | 3 | 0.00060295 | 8.31E-03 |
| GO:0002250 | adaptive immune response | BP | 4 | 0.00065015 | 8.87E-03 |
| GO:0046425 | regulation of JAK-STAT cascade | BP | 3 | 0.00069336 | 9.28E-03 |
| GO:1904892 | regulation of STAT cascade | BP | 3 | 0.00069336 | 9.28E-03 |
| GO:0034622 | cellular macromolecular complex assembly | BP | 6 | 0.00071033 | 9.42E-03 |
| GO:0002703 | regulation of leukocyte mediated immunity | BP | 3 | 0.00074869 | 9.84E-03 |
| GO:0031497 | chromatin assembly | BP | 3 | 0.00083684 | 1.09E-02 |
| GO:0002823 | negative regulation of adaptive immune response based on somatic recombination of immune receptors built from immunoglobulin superfamily domains | CC | 2 | 0.00084794 | 1.09E-02 |
| GO:0044391 | ribosomal subunit | CC | 3 | 0.00086763 | 1.11E-02 |
| GO:0002707 | negative regulation of lymphocyte mediated immunity | BP | 2 | 0.00089439 | 1.13E-02 |
| GO:0022407 | regulation of cell-cell adhesion | BP | 4 | 0.00096265 | 1.21E-02 |
| GO:0034728 | nucleosome organization | BP | 3 | 0.00098101 | 0.01220621 |
| GO:0002820 | negative regulation of adaptive immune response | BP | 2 | 0.0009909 | 0.01222107 |
| GO:0097696 | STAT cascade | BP | 3 | 0.00103234 | 0.01250917 |
| GO:0007259 | JAK-STAT cascade | BP | 3 | 0.00103234 | 0.01250917 |
| GO:0060338 | regulation of type I interferon-mediated signaling pathway | BP | 2 | 0.00104095 | 0.01250917 |
| GO:0071704 | organic substance metabolic process | BP | 22 | 0.00110449 | 0.01309671 |
| GO:0031324 | negative regulation of cellular metabolic process | BP | 9 | 0.00110847 | 0.01309671 |
| GO:0032993 | protein-DNA complex | CC | 3 | 0.00112161 | 0.01311172 |
| GO:0006139 | nucleobase-containing compound metabolic process | BP | 15 | 0.00112839 | 0.01311172 |
| GO:0007155 | cell adhesion | BP | 7 | 0.00118501 | 0.01365672 |
| GO:0006959 | humoral immune response | BP | 3 | 0.00121561 | 0.01382132 |
| GO:0022610 | biological adhesion | BP | 7 | 0.00121895 | 0.01382132 |
| GO:0016337 | single organismal cell-cell adhesion | BP | 5 | 0.00126044 | 0.01417741 |
| GO:0006333 | chromatin assembly or disassembly | BP | 3 | 0.00127433 | 0.01421992 |
| GO:0051704 | multi-organism process | BP | 9 | 0.00133571 | 0.0147875 |
| GO:0043330 | response to exogenous dsRNA | BP | 2 | 0.00136624 | 0.01500725 |
| GO:1901363 | heterocyclic compound binding | MF | 15 | 0.00137785 | 0.01501754 |
| GO:0046483 | heterocycle metabolic process | BP | 15 | 0.00141817 | 0.01533801 |
| GO:0002704 | negative regulation of leukocyte mediated immunity | BP | 2 | 0.00148412 | 0.0159288 |
| GO:0006323 | DNA packaging | BP | 3 | 0.001527 | 0.01609295 |
| GO:0042110 | T cell activation | BP | 4 | 0.00154421 | 0.01609295 |
| GO:0070489 | T cell aggregation | BP | 4 | 0.00154421 | 0.01609295 |
| GO:0006725 | cellular aromatic compound metabolic process | BP | 15 | 0.0015452 | 0.01609295 |
| GO:0071593 | lymphocyte aggregation | BP | 4 | 0.00155733 | 0.01610007 |
| GO:0097159 | organic cyclic compound binding | MF | 15 | 0.00158683 | 0.0162853 |
| GO:0070486 | leukocyte aggregation | BP | 4 | 0.0016514 | 0.01682512 |
| GO:0098602 | single organism cell adhesion | BP | 5 | 0.00169605 | 0.01715574 |
| GO:0002709 | regulation of T cell mediated immunity | BP | 2 | 0.00179931 | 0.01806011 |
| GO:0022607 | cellular component assembly | BP | 9 | 0.00181115 | 0.01806011 |
| GO:0003735 | structural constituent of ribosome | MF | 3 | 0.00183392 | 0.01815821 |
| GO:0009892 | negative regulation of metabolic process | BP | 9 | 0.00184682 | 0.01815821 |
| GO:0042255 | ribosome assembly | BP | 2 | 0.00193352 | 0.01887871 |
| GO:0002449 | lymphocyte mediated immunity | BP | 3 | 0.0020406 | 0.0197868 |
| GO:1901360 | organic cyclic compound metabolic process | BP | 15 | 0.00206548 | 0.01982129 |
| GO:0002718 | regulation of cytokine production involved in immune response | BP | 2 | 0.00207235 | 0.01982129 |
| GO:0007159 | leukocyte cell-cell adhesion | BP | 4 | 0.00211589 | 0.02002076 |
| GO:0065004 | protein-DNA complex assembly | BP | 3 | 0.00212169 | 0.02002076 |
| GO:0044445 | cytosolic part | CC | 3 | 0.00214916 | 0.02014476 |
| GO:0005840 | ribosome | CC | 3 | 0.00217685 | 0.02022979 |
| GO:0007162 | negative regulation of cell adhesion | BP | 3 | 0.00220476 | 0.02022979 |
| GO:0022625 | cytosolic large ribosomal subunit | CC | 2 | 0.00221578 | 0.02022979 |
| GO:0030864 | cortical actin cytoskeleton | CC | 2 | 0.00221578 | 0.02022979 |
| GO:0045087 | innate immune response | BP | 5 | 0.0022825 | 0.02057601 |
| GO:0048523 | negative regulation of cellular process | BP | 12 | 0.00228297 | 0.02057601 |
| GO:0001071 | nucleic acid binding transcription factor activity | MF | 6 | 0.00235758 | 0.02097947 |
| GO:0003700 | transcription factor activity, sequence-specific DNA binding | MF | 6 | 0.00235758 | 0.02097947 |
| GO:0042742 | defense response to bacterium | BP | 3 | 0.0023769 | 0.02101838 |
| GO:0065003 | macromolecular complex assembly | BP | 7 | 0.00248184 | 0.0218092 |
| GO:0050878 | regulation of body fluid levels | BP | 4 | 0.00283966 | 0.02479851 |
| GO:0071824 | protein-DNA complex subunit organization | BP | 3 | 0.00290919 | 0.02524886 |
| GO:0002367 | cytokine production involved in immune response | BP | 2 | 0.00325864 | 0.02810826 |
| GO:0050830 | defense response to Gram-positive bacterium | BP | 2 | 0.0033467 | 0.02869185 |
| GO:0044085 | cellular component biogenesis | BP | 9 | 0.00362241 | 0.03086736 |
| GO:0002456 | T cell mediated immunity | BP | 2 | 0.00371 | 0.03139267 |
| GO:0005576 | extracellular region | CC | 12 | 0.00372872 | 0.03139267 |
| GO:0071103 | DNA conformation change | BP | 3 | 0.00393547 | 0.03283961 |
| GO:0098542 | defense response to other organism | BP | 4 | 0.00396297 | 0.03283961 |
| GO:0071357 | cellular response to type I interferon | BP | 2 | 0.00399401 | 0.03283961 |
| GO:0060337 | type I interferon signaling pathway | BP | 2 | 0.00399401 | 0.03283961 |
| GO:0005126 | cytokine receptor binding | MF | 3 | 0.00409783 | 0.03349735 |
| GO:0030863 | cortical cytoskeleton | CC | 2 | 0.0041888 | 0.0338204 |
| GO:0043331 | response to dsRNA | BP | 2 | 0.0041888 | 0.0338204 |
| GO:0010605 | negative regulation of macromolecule metabolic process | BP | 8 | 0.00420951 | 0.0338204 |
| GO:0048519 | negative regulation of biological process | BP | 12 | 0.00423992 | 0.0338712 |
| GO:0043065 | positive regulation of apoptotic process | BP | 4 | 0.00427161 | 0.03393153 |
| GO:0034340 | response to type I interferon | BP | 2 | 0.00438792 | 0.03440701 |
| GO:0002443 | leukocyte mediated immunity | BP | 3 | 0.00439167 | 0.03440701 |
| GO:0043068 | positive regulation of programmed cell death | BP | 4 | 0.00440488 | 0.03440701 |
| GO:0010033 | response to organic substance | BP | 9 | 0.00444581 | 0.03453486 |
| GO:0050868 | negative regulation of T cell activation | BP | 2 | 0.00459137 | 0.03541285 |
| GO:0050863 | regulation of T cell activation | BP | 3 | 0.00460921 | 0.03541285 |
| GO:0042100 | B cell proliferation | BP | 2 | 0.0046947 | 0.03587366 |
| GO:0002286 | T cell activation involved in immune response | BP | 2 | 0.00479911 | 0.03647324 |
| GO:0000790 | nuclear chromatin | CC | 3 | 0.00483319 | 0.03653473 |
| GO:0033138 | positive regulation of peptidyl-serine phosphorylation | BP | 2 | 0.00490459 | 0.03687621 |
| GO:0046649 | lymphocyte activation | BP | 4 | 0.00514346 | 0.03846648 |
| GO:1903037 | regulation of leukocyte cell-cell adhesion | BP | 3 | 0.00520507 | 0.03872131 |
| GO:1903038 | negative regulation of leukocyte cell-cell adhesion | BP | 2 | 0.00533714 | 0.03928806 |
| GO:0015934 | large ribosomal subunit | CC | 2 | 0.00533714 | 0.03928806 |
| GO:0010942 | positive regulation of cell death | BP | 4 | 0.00538646 | 0.03944461 |
| GO:0030155 | regulation of cell adhesion | BP | 4 | 0.00609424 | 0.04439635 |
| GO:0002700 | regulation of production of molecular mediator of immune response | BP | 2 | 0.00613471 | 0.04446082 |
| GO:0002698 | negative regulation of immune effector process | BP | 2 | 0.00649217 | 0.04681019 |
| GO:0006955 | immune response | BP | 6 | 0.00686738 | 0.0481246 |
| GO:0002457 | T cell antigen processing and presentation | BP | 1 | 0.00691406 | 0.0481246 |
| GO:0070627 | ferrous iron import | BP | 1 | 0.00691406 | 0.0481246 |
| GO:0002578 | negative regulation of antigen processing and presentation | BP | 1 | 0.00691406 | 0.0481246 |
| GO:0000064 | L-ornithine transmembrane transporter activity | MF | 1 | 0.00691406 | 0.0481246 |
| GO:0031088 | platelet dense granule membrane | CC | 1 | 0.00691406 | 0.0481246 |
| GO:1903352 | L-ornithine transmembrane transport | BP | 1 | 0.00691406 | 0.0481246 |

**Supplementary Table S15. GO enrichment analysis of PSGs in the** **Siberian musk deer**.

| **Go ID** | **GO Term** | **GO class** | **Count** | **p-value** | **FDR** |
| --- | --- | --- | --- | --- | --- |
| GO:0044464 | cell part | CC | 160 | 3.97E-40 | 8.80E-37 |
| GO:0005623 | cell | CC | 160 | 5.03E-40 | 8.80E-37 |
| GO:0009987 | cellular process | BP | 149 | 2.33E-34 | 2.21E-31 |
| GO:0005622 | intracellular | CC | 145 | 2.53E-34 | 2.21E-31 |
| GO:0044424 | intracellular part | CC | 141 | 2.22E-32 | 1.55E-29 |
| GO:0005488 | binding | MF | 141 | 1.52E-30 | 8.89E-28 |
| GO:0043226 | organelle | CC | 134 | 9.74E-30 | 4.87E-27 |
| GO:0044699 | single-organism process | BP | 129 | 5.46E-26 | 2.38E-23 |
| GO:0043227 | membrane-bounded organelle | CC | 122 | 1.80E-24 | 7.01E-22 |
| GO:0005737 | cytoplasm | CC | 114 | 5.43E-24 | 1.90E-21 |
| GO:0043229 | intracellular organelle | CC | 117 | 1.11E-21 | 3.52E-19 |
| GO:0003824 | catalytic activity | MF | 79 | 2.38E-21 | 6.93E-19 |
| GO:0044763 | single-organism cellular process | BP | 114 | 1.86E-20 | 5.01E-18 |
| GO:0005515 | protein binding | MF | 109 | 2.56E-20 | 6.38E-18 |
| GO:0044446 | intracellular organelle part | CC | 93 | 4.89E-20 | 1.10E-17 |
| GO:0044238 | primary metabolic process | BP | 104 | 5.03E-20 | 1.10E-17 |
| GO:0044422 | organelle part | CC | 94 | 5.74E-20 | 1.18E-17 |
| GO:0008152 | metabolic process | BP | 108 | 1.18E-19 | 2.29E-17 |
| GO:0071704 | organic substance metabolic process | BP | 104 | 1.61E-18 | 2.96E-16 |
| GO:0043231 | intracellular membrane-bounded organelle | CC | 104 | 2.81E-17 | 4.91E-15 |
| GO:0044237 | cellular metabolic process | BP | 99 | 4.35E-17 | 7.24E-15 |
| GO:0065007 | biological regulation | BP | 105 | 1.59E-16 | 2.54E-14 |
| GO:0043170 | macromolecule metabolic process | BP | 91 | 2.99E-16 | 4.55E-14 |
| GO:0050789 | regulation of biological process | BP | 101 | 3.75E-16 | 5.46E-14 |
| GO:0044444 | cytoplasmic part | CC | 84 | 3.27E-15 | 4.58E-13 |
| GO:0044260 | cellular macromolecule metabolic process | BP | 83 | 4.65E-14 | 6.25E-12 |
| GO:0043232 | intracellular non-membrane-bounded organelle | CC | 54 | 5.29E-14 | 6.61E-12 |
| GO:0043228 | non-membrane-bounded organelle | CC | 54 | 5.29E-14 | 6.61E-12 |
| GO:0050794 | regulation of cellular process | BP | 93 | 1.52E-13 | 1.83E-11 |
| GO:0016787 | hydrolase activity | MF | 41 | 2.97E-13 | 3.47E-11 |
| GO:0070013 | intracellular organelle lumen | CC | 55 | 2.75E-12 | 2.95E-10 |
| GO:0031974 | membrane-enclosed lumen | CC | 55 | 2.78E-12 | 2.95E-10 |
| GO:0043233 | organelle lumen | CC | 55 | 2.78E-12 | 2.95E-10 |
| GO:0071840 | cellular component organization or biogenesis | BP | 66 | 4.14E-12 | 4.25E-10 |
| GO:0016043 | cellular component organization | BP | 65 | 4.25E-12 | 4.25E-10 |
| GO:0046872 | metal ion binding | MF | 52 | 6.57E-12 | 6.38E-10 |
| GO:0043169 | cation binding | MF | 52 | 9.38E-12 | 8.86E-10 |
| GO:0043167 | ion binding | MF | 52 | 3.54E-11 | 3.26E-09 |
| GO:0046483 | heterocycle metabolic process | BP | 61 | 1.30E-10 | 1.17E-08 |
| GO:0006139 | nucleobase-containing compound metabolic process | BP | 60 | 1.59E-10 | 1.39E-08 |
| GO:0006725 | cellular aromatic compound metabolic process | BP | 61 | 1.83E-10 | 1.56E-08 |
| GO:0044428 | nuclear part | CC | 48 | 1.98E-10 | 1.65E-08 |
| GO:0006996 | organelle organization | BP | 46 | 2.08E-10 | 1.69E-08 |
| GO:0034641 | cellular nitrogen compound metabolic process | BP | 64 | 2.18E-10 | 1.73E-08 |
| GO:0019538 | protein metabolic process | BP | 57 | 2.35E-10 | 1.82E-08 |
| GO:0048518 | positive regulation of biological process | BP | 56 | 2.84E-10 | 2.16E-08 |
| GO:0031981 | nuclear lumen | CC | 45 | 3.01E-10 | 2.24E-08 |
| GO:0006807 | nitrogen compound metabolic process | BP | 66 | 4.35E-10 | 3.17E-08 |
| GO:1901360 | organic cyclic compound metabolic process | BP | 61 | 5.74E-10 | 4.10E-08 |
| GO:0043412 | macromolecule modification | BP | 47 | 6.41E-10 | 4.48E-08 |
| GO:0005856 | cytoskeleton | CC | 32 | 8.84E-10 | 6.06E-08 |
| GO:0015630 | microtubule cytoskeleton | CC | 23 | 1.34E-09 | 9.03E-08 |
| GO:0044707 | single-multicellular organism process | BP | 59 | 2.21E-09 | 1.46E-07 |
| GO:0005634 | nucleus | CC | 65 | 2.47E-09 | 1.60E-07 |
| GO:0006464 | cellular protein modification process | BP | 44 | 5.23E-09 | 3.26E-07 |
| GO:0036211 | protein modification process | BP | 44 | 5.23E-09 | 3.26E-07 |
| GO:0044267 | cellular protein metabolic process | BP | 51 | 5.33E-09 | 3.27E-07 |
| GO:0032501 | multicellular organismal process | BP | 64 | 5.74E-09 | 3.46E-07 |
| GO:0043234 | protein complex | CC | 45 | 8.68E-09 | 5.14E-07 |
| GO:0050896 | response to stimulus | BP | 71 | 1.11E-08 | 6.49E-07 |
| GO:0005829 | cytosol | CC | 41 | 1.36E-08 | 7.82E-07 |
| GO:0032991 | macromolecular complex | CC | 49 | 1.88E-08 | 1.06E-06 |
| GO:1901363 | heterocyclic compound binding | MF | 56 | 2.05E-08 | 1.14E-06 |
| GO:0048519 | negative regulation of biological process | BP | 48 | 2.44E-08 | 1.33E-06 |
| GO:0044430 | cytoskeletal part | CC | 25 | 2.76E-08 | 1.48E-06 |
| GO:0005654 | nucleoplasm | CC | 37 | 2.85E-08 | 1.51E-06 |
| GO:0090304 | nucleic acid metabolic process | BP | 51 | 2.91E-08 | 1.52E-06 |
| GO:0097159 | organic cyclic compound binding | MF | 56 | 3.32E-08 | 1.71E-06 |
| GO:0048522 | positive regulation of cellular process | BP | 48 | 3.81E-08 | 1.93E-06 |
| GO:0005576 | extracellular region | CC | 47 | 4.22E-08 | 2.11E-06 |
| GO:0080090 | regulation of primary metabolic process | BP | 54 | 9.03E-08 | 4.45E-06 |
| GO:0060255 | regulation of macromolecule metabolic process | BP | 54 | 9.52E-08 | 4.62E-06 |
| GO:0008270 | zinc ion binding | MF | 21 | 1.20E-07 | 5.75E-06 |
| GO:0046914 | transition metal ion binding | MF | 23 | 1.58E-07 | 7.45E-06 |
| GO:0044700 | single organism signaling | BP | 55 | 2.53E-07 | 1.18E-05 |
| GO:0023052 | signaling | BP | 55 | 2.61E-07 | 1.20E-05 |
| GO:0007154 | cell communication | BP | 55 | 2.94E-07 | 1.34E-05 |
| GO:0031323 | regulation of cellular metabolic process | BP | 53 | 3.12E-07 | 1.40E-05 |
| GO:0051234 | establishment of localization | BP | 46 | 3.20E-07 | 1.42E-05 |
| GO:0032502 | developmental process | BP | 53 | 3.27E-07 | 1.43E-05 |
| GO:0016020 | membrane | CC | 71 | 3.81E-07 | 1.65E-05 |
| GO:0048523 | negative regulation of cellular process | BP | 43 | 4.28E-07 | 1.82E-05 |
| GO:0051179 | localization | BP | 52 | 4.31E-07 | 1.82E-05 |
| GO:0051716 | cellular response to stimulus | BP | 59 | 4.37E-07 | 1.82E-05 |
| GO:0044767 | single-organism developmental process | BP | 52 | 4.89E-07 | 2.01E-05 |
| GO:0019222 | regulation of metabolic process | BP | 54 | 6.69E-07 | 2.72E-05 |
| GO:0016740 | transferase activity | MF | 29 | 7.90E-07 | 3.18E-05 |
| GO:0016887 | ATPase activity | MF | 12 | 8.37E-07 | 3.33E-05 |
| GO:0022402 | cell cycle process | BP | 21 | 8.88E-07 | 3.49E-05 |
| GO:0006810 | transport | BP | 44 | 9.74E-07 | 3.79E-05 |
| GO:0007049 | cell cycle | BP | 24 | 1.04E-06 | 3.98E-05 |
| GO:0005524 | ATP binding | MF | 22 | 1.42E-06 | 5.38E-05 |
| GO:0009653 | anatomical structure morphogenesis | BP | 30 | 1.98E-06 | 7.45E-05 |
| GO:0032559 | adenyl ribonucleotide binding | MF | 22 | 2.12E-06 | 7.87E-05 |
| GO:0030554 | adenyl nucleotide binding | MF | 22 | 2.38E-06 | 8.75E-05 |
| GO:2000113 | negative regulation of cellular macromolecule biosynthetic process | BP | 20 | 3.31E-06 | 0.000120211 |
| GO:0048856 | anatomical structure development | BP | 48 | 3.35E-06 | 0.000120211 |
| GO:0097367 | carbohydrate derivative binding | MF | 27 | 3.37E-06 | 0.000120211 |
| GO:0044421 | extracellular region part | CC | 38 | 3.40E-06 | 0.000120211 |
| GO:1901575 | organic substance catabolic process | BP | 24 | 3.51E-06 | 0.000122699 |
| GO:0034654 | nucleobase-containing compound biosynthetic process | BP | 41 | 3.80E-06 | 0.000131689 |
| GO:0001883 | purine nucleoside binding | MF | 24 | 3.92E-06 | 0.000134517 |
| GO:0009892 | negative regulation of metabolic process | BP | 29 | 3.98E-06 | 0.000135216 |
| GO:0001882 | nucleoside binding | MF | 24 | 4.19E-06 | 0.000140749 |
| GO:0010604 | positive regulation of macromolecule metabolic process | BP | 31 | 4.73E-06 | 0.000157478 |
| GO:0031982 | vesicle | CC | 37 | 4.94E-06 | 0.000161749 |
| GO:0044249 | cellular biosynthetic process | BP | 51 | 4.95E-06 | 0.000161749 |
| GO:0018130 | heterocycle biosynthetic process | BP | 41 | 5.16E-06 | 0.000165754 |
| GO:0070647 | protein modification by small protein conjugation or removal | BP | 17 | 5.17E-06 | 0.000165754 |
| GO:0019438 | aromatic compound biosynthetic process | BP | 41 | 5.56E-06 | 0.000176629 |
| GO:0051172 | negative regulation of nitrogen compound metabolic process | BP | 21 | 6.16E-06 | 0.000194104 |
| GO:0009890 | negative regulation of biosynthetic process | BP | 21 | 6.29E-06 | 0.000196316 |
| GO:0051276 | chromosome organization | BP | 18 | 6.84E-06 | 0.000211579 |
| GO:0016070 | RNA metabolic process | BP | 42 | 6.96E-06 | 0.000213496 |
| GO:2000112 | regulation of cellular macromolecule biosynthetic process | BP | 38 | 7.13E-06 | 0.000216926 |
| GO:2001020 | regulation of response to DNA damage stimulus | BP | 7 | 7.23E-06 | 0.000218035 |
| GO:0010605 | negative regulation of macromolecule metabolic process | BP | 27 | 7.30E-06 | 0.000218181 |
| GO:0003676 | nucleic acid binding | MF | 38 | 8.41E-06 | 0.000249169 |
| GO:0031324 | negative regulation of cellular metabolic process | BP | 27 | 8.60E-06 | 0.000251464 |
| GO:0009056 | catabolic process | BP | 24 | 8.63E-06 | 0.000251464 |
| GO:0010558 | negative regulation of macromolecule biosynthetic process | BP | 20 | 8.94E-06 | 0.000258272 |
| GO:0005815 | microtubule organizing center | CC | 13 | 9.17E-06 | 0.00026281 |
| GO:0044451 | nucleoplasm part | CC | 14 | 9.26E-06 | 0.000263166 |
| GO:0042995 | cell projection | CC | 23 | 9.65E-06 | 0.000272179 |
| GO:1901362 | organic cyclic compound biosynthetic process | BP | 41 | 9.73E-06 | 0.000272316 |
| GO:0007165 | signal transduction | BP | 48 | 1.06E-05 | 0.000293084 |
| GO:0035639 | purine ribonucleoside triphosphate binding | MF | 23 | 1.11E-05 | 0.000306224 |
| GO:0032550 | purine ribonucleoside binding | MF | 23 | 1.21E-05 | 0.000330962 |
| GO:0031325 | positive regulation of cellular metabolic process | BP | 30 | 1.22E-05 | 0.000330962 |
| GO:0032549 | ribonucleoside binding | MF | 23 | 1.25E-05 | 0.000335226 |
| GO:0009058 | biosynthetic process | BP | 51 | 1.27E-05 | 0.00034015 |
| GO:0036094 | small molecule binding | MF | 28 | 1.33E-05 | 0.000351697 |
| GO:0043933 | macromolecular complex subunit organization | BP | 27 | 1.34E-05 | 0.000351697 |
| GO:0007275 | multicellular organism development | BP | 43 | 1.35E-05 | 0.000351697 |
| GO:0010556 | regulation of macromolecule biosynthetic process | BP | 38 | 1.37E-05 | 0.000354332 |
| GO:0031988 | membrane-bounded vesicle | CC | 35 | 1.40E-05 | 0.000359669 |
| GO:0006508 | proteolysis | CC | 21 | 1.41E-05 | 0.000360802 |
| GO:0032555 | purine ribonucleotide binding | MF | 23 | 1.60E-05 | 0.000405354 |
| GO:2001022 | positive regulation of response to DNA damage stimulus | BP | 5 | 1.62E-05 | 0.000407157 |
| GO:0031327 | negative regulation of cellular biosynthetic process | BP | 20 | 1.64E-05 | 0.00041087 |
| GO:0034645 | cellular macromolecule biosynthetic process | BP | 43 | 1.74E-05 | 0.000430792 |
| GO:1901576 | organic substance biosynthetic process | BP | 50 | 1.78E-05 | 0.00043556 |
| GO:0017076 | purine nucleotide binding | MF | 23 | 1.79E-05 | 0.00043556 |
| GO:0009893 | positive regulation of metabolic process | BP | 31 | 1.79E-05 | 0.00043556 |
| GO:0032553 | ribonucleotide binding | MF | 23 | 1.83E-05 | 0.000441672 |
| GO:0034470 | ncRNA processing | BP | 10 | 1.89E-05 | 0.00045262 |
| GO:0048731 | system development | BP | 39 | 2.02E-05 | 0.000478441 |
| GO:0045934 | negative regulation of nucleobase-containing compound metabolic process | BP | 19 | 2.02E-05 | 0.000478441 |
| GO:0031224 | intrinsic component of membrane | CC | 46 | 2.12E-05 | 0.000497399 |
| GO:0005813 | centrosome | CC | 11 | 2.13E-05 | 0.000497688 |
| GO:1902578 | single-organism localization | BP | 30 | 2.41E-05 | 0.000558727 |
| GO:0051239 | regulation of multicellular organismal process | BP | 28 | 2.58E-05 | 0.000593564 |
| GO:0051171 | regulation of nitrogen compound metabolic process | BP | 39 | 2.74E-05 | 0.000625812 |
| GO:0070011 | peptidase activity, acting on L-amino acid peptides | MF | 12 | 2.87E-05 | 0.000651675 |
| GO:0000166 | nucleotide binding | MF | 26 | 2.90E-05 | 0.000655027 |
| GO:1901265 | nucleoside phosphate binding | MF | 26 | 2.92E-05 | 0.000655532 |
| GO:0044710 | single-organism metabolic process | BP | 37 | 2.97E-05 | 0.000658986 |
| GO:0044248 | cellular catabolic process | BP | 20 | 2.98E-05 | 0.000658986 |
| GO:0019219 | regulation of nucleobase-containing compound metabolic process | BP | 37 | 3.13E-05 | 0.000687657 |
| GO:0006950 | response to stress | BP | 35 | 3.15E-05 | 0.000687657 |
| GO:0035637 | multicellular organismal signaling | BP | 7 | 3.28E-05 | 0.000711505 |
| GO:0044271 | cellular nitrogen compound biosynthetic process | BP | 42 | 3.44E-05 | 0.000742496 |
| GO:0031326 | regulation of cellular biosynthetic process | BP | 38 | 3.60E-05 | 0.000771819 |
| GO:0016570 | histone modification | BP | 10 | 3.62E-05 | 0.000771819 |
| GO:0009059 | macromolecule biosynthetic process | BP | 43 | 3.81E-05 | 0.000807197 |
| GO:0006811 | ion transport | BP | 19 | 3.87E-05 | 0.00081517 |
| GO:0008233 | peptidase activity | MF | 12 | 3.92E-05 | 0.000820841 |
| GO:0006812 | cation transport | BP | 15 | 4.70E-05 | 0.000977528 |
| GO:0016569 | covalent chromatin modification | BP | 10 | 4.72E-05 | 0.000977531 |
| GO:0009889 | regulation of biosynthetic process | BP | 38 | 4.78E-05 | 0.000977562 |
| GO:0006325 | chromatin organization | BP | 13 | 4.78E-05 | 0.000977562 |
| GO:0008134 | transcription factor binding | MF | 11 | 4.84E-05 | 0.000984973 |
| GO:1902589 | single-organism organelle organization | BP | 21 | 4.99E-05 | 0.001007858 |
| GO:0008757 | S-adenosylmethionine-dependent methyltransferase activity | MF | 6 | 5.36E-05 | 0.001077768 |
| GO:0010629 | negative regulation of gene expression | BP | 19 | 5.43E-05 | 0.001085653 |
| GO:0008168 | methyltransferase activity | MF | 7 | 5.78E-05 | 0.001148065 |
| GO:0023051 | regulation of signaling | BP | 30 | 5.95E-05 | 0.00117573 |
| GO:0006796 | phosphate-containing compound metabolic process | BP | 30 | 6.02E-05 | 0.001183174 |
| GO:0012505 | endomembrane system | CC | 35 | 6.31E-05 | 0.001227128 |
| GO:0006793 | phosphorus metabolic process | BP | 30 | 6.32E-05 | 0.001227128 |
| GO:0005819 | spindle | CC | 8 | 6.82E-05 | 0.001316953 |
| GO:0006355 | regulation of transcription, DNA-templated | BP | 33 | 6.85E-05 | 0.001316953 |
| GO:0035556 | intracellular signal transduction | BP | 27 | 7.52E-05 | 0.00143629 |
| GO:1903506 | regulation of nucleic acid-templated transcription | BP | 33 | 7.60E-05 | 0.001444647 |
| GO:1903561 | extracellular vesicle | CC | 28 | 7.77E-05 | 0.001469638 |
| GO:0043230 | extracellular organelle | CC | 28 | 7.82E-05 | 0.001470766 |
| GO:0016741 | transferase activity, transferring one-carbon groups | MF | 7 | 8.19E-05 | 0.001530767 |
| GO:2001141 | regulation of RNA biosynthetic process | BP | 33 | 8.38E-05 | 0.001558123 |
| GO:0010467 | gene expression | BP | 43 | 9.51E-05 | 0.001759702 |
| GO:0007017 | microtubule-based process | BP | 11 | 9.81E-05 | 0.001805659 |
| GO:0030001 | metal ion transport | BP | 13 | 0.00010617 | 0.001943412 |
| GO:0044765 | single-organism transport | BP | 27 | 0.0001067 | 0.001943412 |
| GO:0007010 | cytoskeleton organization | BP | 15 | 0.00011685 | 0.002117267 |
| GO:0016021 | integral component of membrane | CC | 43 | 0.00011866 | 0.002136588 |
| GO:0000226 | microtubule cytoskeleton organization | BP | 9 | 0.00011914 | 0.002136588 |
| GO:0044425 | membrane part | CC | 50 | 0.00012282 | 0.00219138 |
| GO:0008324 | cation transmembrane transporter activity | MF | 11 | 0.0001295 | 0.002295171 |
| GO:0033554 | cellular response to stress | BP | 21 | 0.00012995 | 0.002295171 |
| GO:0008092 | cytoskeletal protein binding | MF | 13 | 0.00013662 | 0.002400784 |
| GO:0022853 | active ion transmembrane transporter activity | MF | 5 | 0.00014279 | 0.002496686 |
| GO:0022890 | inorganic cation transmembrane transporter activity | MF | 10 | 0.000144 | 0.002505343 |
| GO:0051252 | regulation of RNA metabolic process | BP | 33 | 0.00014885 | 0.002576833 |
| GO:0005730 | nucleolus | CC | 13 | 0.00015719 | 0.002697925 |
| GO:0006351 | transcription, DNA-templated | BP | 33 | 0.00015739 | 0.002697925 |
| GO:0071944 | cell periphery | CC | 41 | 0.00015846 | 0.002701593 |
| GO:0046873 | metal ion transmembrane transporter activity | MF | 9 | 0.00015914 | 0.002701593 |
| GO:0006928 | movement of cell or subcellular component | BP | 20 | 0.00016037 | 0.002709194 |
| GO:0044085 | cellular component biogenesis | BP | 27 | 0.00016257 | 0.002733282 |
| GO:0097659 | nucleic acid-templated transcription | BP | 33 | 0.00016889 | 0.002825863 |
| GO:0071702 | organic substance transport | BP | 26 | 0.00017334 | 0.002886582 |
| GO:0070062 | extracellular exosome | CC | 27 | 0.00017648 | 0.002924806 |
| GO:0017111 | nucleoside-triphosphatase activity | MF | 12 | 0.00019887 | 0.003280414 |
| GO:0008033 | tRNA processing | BP | 5 | 0.00021686 | 0.003560379 |
| GO:0010468 | regulation of gene expression | BP | 36 | 0.00022812 | 0.003727657 |
| GO:1903047 | mitotic cell cycle process | BP | 13 | 0.00024088 | 0.00391794 |
| GO:0045892 | negative regulation of transcription, DNA-templated | BP | 15 | 0.00024531 | 0.003971464 |
| GO:0010646 | regulation of cell communication | BP | 28 | 0.00025375 | 0.004089296 |
| GO:0008237 | metallopeptidase activity | MF | 6 | 0.0002563 | 0.004111442 |
| GO:0000027 | ribosomal large subunit assembly | BP | 3 | 0.00025926 | 0.00413995 |
| GO:0009628 | response to abiotic stimulus | BP | 15 | 0.00027189 | 0.004321778 |
| GO:0044459 | plasma membrane part | CC | 25 | 0.00028008 | 0.004431879 |
| GO:0048646 | anatomical structure formation involved in morphogenesis | BP | 15 | 0.00029007 | 0.004569237 |
| GO:0034660 | ncRNA metabolic process | BP | 10 | 0.00029434 | 0.004615713 |
| GO:0032774 | RNA biosynthetic process | BP | 33 | 0.00030458 | 0.004754938 |
| GO:0006974 | cellular response to DNA damage stimulus | BP | 12 | 0.00031494 | 0.004894923 |
| GO:0016462 | pyrophosphatase activity | MF | 12 | 0.00031855 | 0.004929054 |
| GO:0080135 | regulation of cellular response to stress | BP | 10 | 0.00032008 | 0.00493096 |
| GO:0048858 | cell projection morphogenesis | BP | 12 | 0.00032587 | 0.004975924 |
| GO:0016818 | hydrolase activity, acting on acid anhydrides, in phosphorus-containing anhydrides | MF | 12 | 0.00032587 | 0.004975924 |
| GO:0015031 | protein transport | BP | 20 | 0.00032727 | 0.004975924 |
| GO:0006302 | double-strand break repair | BP | 6 | 0.00032869 | 0.00497595 |
| GO:0016817 | hydrolase activity, acting on acid anhydrides | MF | 12 | 0.00033332 | 0.005024282 |
| GO:1903507 | negative regulation of nucleic acid-templated transcription | BP | 15 | 0.0003606 | 0.005412155 |
| GO:0016579 | protein deubiquitination | BP | 5 | 0.00037873 | 0.005659946 |
| GO:0015075 | ion transmembrane transporter activity | MF | 12 | 0.00038542 | 0.005723968 |
| GO:0098655 | cation transmembrane transport | BP | 11 | 0.00038629 | 0.005723968 |
| GO:0048869 | cellular developmental process | BP | 33 | 0.00039185 | 0.005775419 |
| GO:0048147 | negative regulation of fibroblast proliferation | BP | 3 | 0.00039307 | 0.005775419 |
| GO:0032990 | cell part morphogenesis | BP | 12 | 0.00040722 | 0.005958344 |
| GO:0022891 | substrate-specific transmembrane transporter activity | MF | 13 | 0.00041116 | 0.005990924 |
| GO:1902679 | negative regulation of RNA biosynthetic process | BP | 15 | 0.00041908 | 0.00608106 |
| GO:0005886 | plasma membrane | CC | 39 | 0.00042646 | 0.006162549 |
| GO:0032436 | positive regulation of proteasomal ubiquitin-dependent protein catabolic process | BP | 4 | 0.00043161 | 0.006211233 |
| GO:0015379 | potassium:chloride symporter activity | MF | 2 | 0.00043651 | 0.006230515 |
| GO:0022820 | potassium ion symporter activity | MF | 2 | 0.00043651 | 0.006230515 |
| GO:0070925 | organelle assembly | BP | 10 | 0.00044852 | 0.006375888 |
| GO:0022607 | cellular component assembly | BP | 24 | 0.00047936 | 0.006786701 |
| GO:0048583 | regulation of response to stimulus | BP | 31 | 0.00049057 | 0.006917398 |
| GO:0000278 | mitotic cell cycle | BP | 13 | 0.00049663 | 0.006974823 |
| GO:0006282 | regulation of DNA repair | BP | 4 | 0.00050006 | 0.006987343 |
| GO:0034220 | ion transmembrane transport | BP | 13 | 0.00050152 | 0.006987343 |
| GO:0098660 | inorganic ion transmembrane transport | BP | 11 | 0.00051321 | 0.007121773 |
| GO:0031624 | ubiquitin conjugating enzyme binding | MF | 3 | 0.00051799 | 0.007159672 |
| GO:0019899 | enzyme binding | MF | 19 | 0.00053463 | 0.007360607 |
| GO:0006813 | potassium ion transport | BP | 6 | 0.00054677 | 0.007498226 |
| GO:0016788 | hydrolase activity, acting on ester bonds | MF | 11 | 0.00056259 | 0.007685008 |
| GO:0007018 | microtubule-based movement | BP | 6 | 0.00058737 | 0.007992368 |
| GO:0051253 | negative regulation of RNA metabolic process | BP | 15 | 0.00059956 | 0.008101397 |
| GO:0031226 | intrinsic component of plasma membrane | CC | 18 | 0.00060002 | 0.008101397 |
| GO:0061136 | regulation of proteasomal protein catabolic process | BP | 5 | 0.00061859 | 0.008320093 |
| GO:0022892 | substrate-specific transporter activity | MF | 14 | 0.00064067 | 0.008583965 |
| GO:0048585 | negative regulation of response to stimulus | BP | 16 | 0.00064944 | 0.008668244 |
| GO:0070646 | protein modification by small protein removal | BP | 5 | 0.00065758 | 0.008710433 |
| GO:0005875 | microtubule associated complex | CC | 5 | 0.00065758 | 0.008710433 |
| GO:0044390 | ubiquitin-like protein conjugating enzyme binding | MF | 3 | 0.0006658 | 0.008720191 |
| GO:0019239 | deaminase activity | MF | 3 | 0.0006658 | 0.008720191 |
| GO:0009225 | nucleotide-sugar metabolic process | BP | 3 | 0.0006658 | 0.008720191 |
| GO:0044057 | regulation of system process | BP | 9 | 0.00068685 | 0.008962402 |
| GO:0010212 | response to ionizing radiation | BP | 5 | 0.00069838 | 0.009078877 |
| GO:0098662 | inorganic cation transmembrane transport | BP | 10 | 0.00070649 | 0.009138718 |
| GO:0042623 | ATPase activity, coupled | MF | 7 | 0.0007082 | 0.009138718 |
| GO:0008156 | negative regulation of DNA replication | BP | 3 | 0.00072043 | 0.00922831 |
| GO:0060795 | cell fate commitment involved in formation of primary germ layer | BP | 3 | 0.00072043 | 0.00922831 |
| GO:0008104 | protein localization | BP | 23 | 0.00073928 | 0.009389796 |
| GO:0070775 | H3 histone acetyltransferase complex | CC | 2 | 0.00074377 | 0.009389796 |
| GO:0016593 | Cdc73/Paf1 complex | CC | 2 | 0.00074377 | 0.009389796 |
| GO:0070776 | MOZ/MORF histone acetyltransferase complex | CC | 2 | 0.00074377 | 0.009389796 |
| GO:0032989 | cellular component morphogenesis | BP | 15 | 0.00081639 | 0.01024843 |
| GO:1901800 | positive regulation of proteasomal protein catabolic process | BP | 4 | 0.00081765 | 0.01024843 |
| GO:0022857 | transmembrane transporter activity | MF | 13 | 0.00084115 | 0.010505321 |
| GO:0000123 | histone acetyltransferase complex | CC | 4 | 0.00085219 | 0.0106054 |
| GO:0032446 | protein modification by small protein conjugation | BP | 12 | 0.00085656 | 0.010622007 |
| GO:0007399 | nervous system development | BP | 21 | 0.00087835 | 0.010829596 |
| GO:0032259 | methylation | BP | 7 | 0.0008795 | 0.010829596 |
| GO:0032403 | protein complex binding | MF | 11 | 0.00090114 | 0.011057202 |
| GO:0005215 | transporter activity | MF | 15 | 0.00091868 | 0.011232936 |
| GO:0015377 | cation:chloride symporter activity | MF | 2 | 0.0009269 | 0.011260407 |
| GO:0033036 | macromolecule localization | BP | 25 | 0.00092737 | 0.011260407 |
| GO:0035257 | nuclear hormone receptor binding | MF | 5 | 0.00093139 | 0.011270141 |
| GO:0045184 | establishment of protein localization | BP | 20 | 0.00095117 | 0.011446229 |
| GO:0005694 | chromosome | CC | 12 | 0.00095249 | 0.011446229 |
| GO:0046129 | purine ribonucleoside biosynthetic process | BP | 4 | 0.00096199 | 0.011481526 |
| GO:0042451 | purine nucleoside biosynthetic process | BP | 4 | 0.00096199 | 0.011481526 |
| GO:0004180 | carboxypeptidase activity | MF | 3 | 0.00096722 | 0.011504668 |
| GO:0007569 | cell aging | BP | 4 | 0.0010007 | 0.011862549 |
| GO:0055085 | transmembrane transport | BP | 15 | 0.00101593 | 0.012002443 |
| GO:0051246 | regulation of protein metabolic process | BP | 23 | 0.00102759 | 0.012099249 |
| GO:1901658 | glycosyl compound catabolic process | BP | 3 | 0.00103626 | 0.012160383 |
| GO:0009057 | macromolecule catabolic process | BP | 14 | 0.00105467 | 0.012314441 |
| GO:0032268 | regulation of cellular protein metabolic process | BP | 22 | 0.00105643 | 0.012314441 |
| GO:0097458 | neuron part | CC | 15 | 0.00106368 | 0.012357779 |
| GO:0005887 | integral component of plasma membrane | CC | 17 | 0.00107152 | 0.012407583 |
| GO:0042493 | response to drug | BP | 8 | 0.00108589 | 0.012532478 |
| GO:0016458 | gene silencing | BP | 6 | 0.00108964 | 0.01253439 |
| GO:0005522 | profilin binding | MF | 2 | 0.00112945 | 0.012907417 |
| GO:0035385 | Roundabout signaling pathway | BP | 2 | 0.00112945 | 0.012907417 |
| GO:0044427 | chromosomal part | CC | 11 | 0.00116582 | 0.013279763 |
| GO:0016605 | PML body | CC | 4 | 0.0012108 | 0.013747267 |
| GO:0000209 | protein polyubiquitination | BP | 6 | 0.00123067 | 0.013927637 |
| GO:0000902 | cell morphogenesis | BP | 14 | 0.00124818 | 0.014080254 |
| GO:0015077 | monovalent inorganic cation transmembrane transporter activity | MF | 7 | 0.00125648 | 0.014128346 |
| GO:0003723 | RNA binding | MF | 17 | 0.00129386 | 0.01445519 |
| GO:0031248 | protein acetyltransferase complex | CC | 4 | 0.00130286 | 0.01445519 |
| GO:0008238 | exopeptidase activity | MF | 4 | 0.00130286 | 0.01445519 |
| GO:1902493 | acetyltransferase complex | CC | 4 | 0.00130286 | 0.01445519 |
| GO:0040029 | regulation of gene expression, epigenetic | BP | 6 | 0.00130622 | 0.01445519 |
| GO:0034703 | cation channel complex | CC | 5 | 0.00131521 | 0.014508788 |
| GO:0045935 | positive regulation of nucleobase-containing compound metabolic process | BP | 17 | 0.00133742 | 0.014674698 |
| GO:0045171 | intercellular bridge | CC | 3 | 0.0013433 | 0.014674698 |
| GO:1901136 | carbohydrate derivative catabolic process | BP | 5 | 0.00134891 | 0.014674698 |
| GO:0032688 | negative regulation of interferon-beta production | BP | 2 | 0.00135123 | 0.014674698 |
| GO:0046085 | adenosine metabolic process | BP | 2 | 0.00135123 | 0.014674698 |
| GO:0044265 | cellular macromolecule catabolic process | BP | 12 | 0.00135551 | 0.014675588 |
| GO:0072331 | signal transduction by p53 class mediator | BP | 6 | 0.00138526 | 0.014951427 |
| GO:0022804 | active transmembrane transporter activity | MF | 7 | 0.00140689 | 0.015091739 |
| GO:0016604 | nuclear body | CC | 7 | 0.00140689 | 0.015091739 |
| GO:0006820 | anion transport | BP | 8 | 0.00141672 | 0.015150634 |
| GO:0007622 | rhythmic behavior | BP | 3 | 0.00142803 | 0.015217863 |
| GO:0009966 | regulation of signal transduction | BP | 24 | 0.00143171 | 0.015217863 |
| GO:0006399 | tRNA metabolic process | BP | 5 | 0.00149014 | 0.015791006 |
| GO:0044712 | single-organism catabolic process | BP | 11 | 0.00150603 | 0.015911114 |
| GO:0034622 | cellular macromolecular complex assembly | BP | 12 | 0.00153574 | 0.016176134 |
| GO:0065003 | macromolecular complex assembly | BP | 17 | 0.00156429 | 0.016285011 |
| GO:0051427 | hormone receptor binding | MF | 5 | 0.0015647 | 0.016285011 |
| GO:0071805 | potassium ion transmembrane transport | BP | 5 | 0.0015647 | 0.016285011 |
| GO:0071804 | cellular potassium ion transport | BP | 5 | 0.0015647 | 0.016285011 |
| GO:0046903 | secretion | BP | 13 | 0.00157378 | 0.016286929 |
| GO:0080134 | regulation of response to stress | BP | 15 | 0.0015742 | 0.016286929 |
| GO:0050793 | regulation of developmental process | BP | 21 | 0.0015907 | 0.016326942 |
| GO:0070525 | tRNA threonylcarbamoyladenosine metabolic process | BP | 2 | 0.00159208 | 0.016326942 |
| GO:0004000 | adenosine deaminase activity | MF | 2 | 0.00159208 | 0.016326942 |
| GO:0003008 | system process | BP | 19 | 0.00161982 | 0.01652207 |
| GO:0042578 | phosphoric ester hydrolase activity | MF | 7 | 0.00162055 | 0.01652207 |
| GO:1990234 | transferase complex | CC | 10 | 0.00165951 | 0.016870049 |
| GO:0030030 | cell projection organization | BP | 14 | 0.00179119 | 0.01810342 |
| GO:0072523 | purine-containing compound catabolic process | BP | 3 | 0.00180022 | 0.01814232 |
| GO:0051254 | positive regulation of RNA metabolic process | BP | 15 | 0.0018396 | 0.018190308 |
| GO:0042127 | regulation of cell proliferation | BP | 16 | 0.00184729 | 0.018190308 |
| GO:0051382 | kinetochore assembly | BP | 2 | 0.0018518 | 0.018190308 |
| GO:0043517 | positive regulation of DNA damage response, signal transduction by p53 class mediator | BP | 2 | 0.0018518 | 0.018190308 |
| GO:0090399 | replicative senescence | BP | 2 | 0.0018518 | 0.018190308 |
| GO:2000780 | negative regulation of double-strand break repair | BP | 2 | 0.0018518 | 0.018190308 |
| GO:0006152 | purine nucleoside catabolic process | BP | 2 | 0.0018518 | 0.018190308 |
| GO:0043194 | axon initial segment | CC | 2 | 0.0018518 | 0.018190308 |
| GO:0046130 | purine ribonucleoside catabolic process | BP | 2 | 0.0018518 | 0.018190308 |
| GO:0042455 | ribonucleoside biosynthetic process | BP | 4 | 0.00189821 | 0.018541966 |
| GO:0032434 | regulation of proteasomal ubiquitin-dependent protein catabolic process | BP | 4 | 0.00189821 | 0.018541966 |
| GO:0015631 | tubulin binding | MF | 6 | 0.00190435 | 0.018550157 |
| GO:0006816 | calcium ion transport | BP | 7 | 0.00200221 | 0.019449265 |
| GO:0065008 | regulation of biological quality | BP | 28 | 0.00202572 | 0.019623094 |
| GO:0034508 | centromere complex assembly | BP | 3 | 0.00211557 | 0.020353606 |
| GO:0016042 | lipid catabolic process | BP | 6 | 0.00211885 | 0.020353606 |
| GO:0048642 | negative regulation of skeletal muscle tissue development | BP | 2 | 0.00213023 | 0.020353606 |
| GO:0030898 | actin-dependent ATPase activity | MF | 2 | 0.00213023 | 0.020353606 |
| GO:0051053 | negative regulation of DNA metabolic process | BP | 4 | 0.00215476 | 0.020531845 |
| GO:0090398 | cellular senescence | BP | 3 | 0.0022278 | 0.021170182 |
| GO:0050680 | negative regulation of epithelial cell proliferation | BP | 4 | 0.00229159 | 0.021717294 |
| GO:0042255 | ribosome assembly | BP | 3 | 0.00234367 | 0.022091118 |
| GO:0072686 | mitotic spindle | CC | 3 | 0.00234367 | 0.022091118 |
| GO:0051383 | kinetochore organization | BP | 2 | 0.00242719 | 0.022755717 |
| GO:0045738 | negative regulation of DNA repair | BP | 2 | 0.00242719 | 0.022755717 |
| GO:0015672 | monovalent inorganic cation transport | BP | 8 | 0.00246239 | 0.023023885 |
| GO:0098656 | anion transmembrane transport | BP | 5 | 0.00246896 | 0.023023885 |
| GO:0051603 | proteolysis involved in cellular protein catabolic process | BP | 9 | 0.00250638 | 0.023310689 |
| GO:0051173 | positive regulation of nitrogen compound metabolic process | BP | 17 | 0.00256945 | 0.023833876 |
| GO:0009163 | nucleoside biosynthetic process | BP | 4 | 0.00258298 | 0.023896006 |
| GO:0018205 | peptidyl-lysine modification | BP | 7 | 0.00262747 | 0.024217852 |
| GO:0008509 | anion transmembrane transporter activity | MF | 5 | 0.00263162 | 0.024217852 |
| GO:0006281 | DNA repair | BP | 8 | 0.00267856 | 0.024585106 |
| GO:0007568 | aging | BP | 6 | 0.00273485 | 0.02491046 |
| GO:1901659 | glycosyl compound biosynthetic process | BP | 4 | 0.0027378 | 0.02491046 |
| GO:0033268 | node of Ranvier | CC | 2 | 0.0027425 | 0.02491046 |
| GO:0043101 | purine-containing compound salvage | BP | 2 | 0.0027425 | 0.02491046 |
| GO:0008017 | microtubule binding | MF | 5 | 0.00280192 | 0.025384214 |
| GO:0042273 | ribosomal large subunit biogenesis | BP | 3 | 0.00284419 | 0.02570063 |
| GO:0016874 | ligase activity | MF | 7 | 0.00285319 | 0.025715469 |
| GO:0001666 | response to hypoxia | BP | 6 | 0.00287263 | 0.025824171 |
| GO:1903522 | regulation of blood circulation | BP | 6 | 0.0029197 | 0.026179961 |
| GO:0009888 | tissue development | BP | 17 | 0.0029599 | 0.026472602 |
| GO:0006400 | tRNA modification | BP | 3 | 0.00297878 | 0.026573457 |
| GO:0016567 | protein ubiquitination | BP | 10 | 0.00304764 | 0.02709508 |
| GO:0006396 | RNA processing | BP | 11 | 0.00306878 | 0.02709508 |
| GO:1903206 | negative regulation of hydrogen peroxide-induced cell death | BP | 2 | 0.00307599 | 0.02709508 |
| GO:1901032 | negative regulation of response to reactive oxygen species | BP | 2 | 0.00307599 | 0.02709508 |
| GO:1901798 | positive regulation of signal transduction by p53 class mediator | BP | 2 | 0.00307599 | 0.02709508 |
| GO:0043966 | histone H3 acetylation | BP | 3 | 0.00311722 | 0.027389265 |
| GO:0045893 | positive regulation of transcription, DNA-templated | BP | 14 | 0.00320522 | 0.028021603 |
| GO:1903508 | positive regulation of nucleic acid-templated transcription | BP | 14 | 0.00320522 | 0.028021603 |
| GO:0003774 | motor activity | MF | 4 | 0.00332959 | 0.029036354 |
| GO:0036293 | response to decreased oxygen levels | BP | 6 | 0.00336966 | 0.029312704 |
| GO:0043543 | protein acylation | BP | 5 | 0.00342731 | 0.029668165 |
| GO:0035561 | regulation of chromatin binding | BP | 2 | 0.00342749 | 0.029668165 |
| GO:1902495 | transmembrane transporter complex | CC | 6 | 0.0034763 | 0.030016321 |
| GO:0030163 | protein catabolic process | BP | 10 | 0.00349409 | 0.030095645 |
| GO:0044257 | cellular protein catabolic process | BP | 9 | 0.00352151 | 0.030257313 |
| GO:0005102 | receptor binding | MF | 15 | 0.00354134 | 0.030353086 |
| GO:0051128 | regulation of cellular component organization | BP | 20 | 0.00357978 | 0.030607532 |
| GO:0061337 | cardiac conduction | BP | 4 | 0.0036079 | 0.030753183 |
| GO:1902680 | positive regulation of RNA biosynthetic process | BP | 14 | 0.0036144 | 0.030753183 |
| GO:0042254 | ribosome biogenesis | BP | 6 | 0.00369708 | 0.031380298 |
| GO:0070838 | divalent metal ion transport | BP | 7 | 0.00376208 | 0.031854673 |
| GO:0065004 | protein-DNA complex assembly | BP | 5 | 0.00377501 | 0.031887011 |
| GO:0050687 | negative regulation of defense response to virus | BP | 2 | 0.00379683 | 0.031993987 |
| GO:1990351 | transporter complex | CC | 6 | 0.0038113 | 0.032038701 |
| GO:0006635 | fatty acid beta-oxidation | BP | 3 | 0.00386846 | 0.032441282 |
| GO:0072511 | divalent inorganic cation transport | BP | 7 | 0.00390803 | 0.032694727 |
| GO:0051049 | regulation of transport | BP | 17 | 0.00395541 | 0.033012103 |
| GO:0010648 | negative regulation of cell communication | BP | 13 | 0.00399148 | 0.033210753 |
| GO:0071456 | cellular response to hypoxia | BP | 4 | 0.004003 | 0.033210753 |
| GO:0007067 | mitotic nuclear division | BP | 7 | 0.0040077 | 0.033210753 |
| GO:0016459 | myosin complex | CC | 3 | 0.00403075 | 0.03332275 |
| GO:0023057 | negative regulation of signaling | BP | 13 | 0.00410297 | 0.03381506 |
| GO:0032879 | regulation of localization | BP | 21 | 0.00410964 | 0.03381506 |
| GO:0048812 | neuron projection morphogenesis | BP | 8 | 0.00412349 | 0.033849391 |
| GO:0006357 | regulation of transcription from RNA polymerase II promoter | BP | 17 | 0.00413417 | 0.033857585 |
| GO:0009226 | nucleotide-sugar biosynthetic process | BP | 2 | 0.00418383 | 0.034025232 |
| GO:1903205 | regulation of hydrogen peroxide-induced cell death | BP | 2 | 0.00418383 | 0.034025232 |
| GO:0046965 | retinoid X receptor binding | MF | 2 | 0.00418383 | 0.034025232 |
| GO:0008016 | regulation of heart contraction | BP | 5 | 0.00422479 | 0.034199313 |
| GO:1903050 | regulation of proteolysis involved in cellular protein catabolic process | BP | 5 | 0.00422479 | 0.034199313 |
| GO:0019226 | transmission of nerve impulse | BP | 3 | 0.0043676 | 0.035273692 |
| GO:0007610 | behavior | BP | 8 | 0.004398 | 0.035437309 |
| GO:0006511 | ubiquitin-dependent protein catabolic process | BP | 8 | 0.00444508 | 0.035734383 |
| GO:0010959 | regulation of metal ion transport | BP | 6 | 0.00448688 | 0.035905319 |
| GO:0070482 | response to oxygen levels | BP | 6 | 0.00448688 | 0.035905319 |
| GO:0031175 | neuron projection development | BP | 10 | 0.00458134 | 0.036577513 |
| GO:0003677 | DNA binding | MF | 21 | 0.00459468 | 0.036600451 |
| GO:0048513 | animal organ development | BP | 25 | 0.00464253 | 0.036897542 |
| GO:0042625 | ATPase coupled ion transmembrane transporter activity | MF | 3 | 0.00472104 | 0.037351756 |
| GO:0007422 | peripheral nervous system development | BP | 3 | 0.00472104 | 0.037351756 |
| GO:0012501 | programmed cell death | BP | 17 | 0.00476181 | 0.037589255 |
| GO:1902580 | single-organism cellular localization | BP | 12 | 0.00481654 | 0.037935652 |
| GO:0019941 | modification-dependent protein catabolic process | BP | 8 | 0.0048359 | 0.03800257 |
| GO:0036294 | cellular response to decreased oxygen levels | BP | 4 | 0.00487895 | 0.038143439 |
| GO:0015079 | potassium ion transmembrane transporter activity | MF | 4 | 0.00487895 | 0.038143439 |
| GO:0043269 | regulation of ion transport | BP | 8 | 0.00488655 | 0.038143439 |
| GO:0070271 | protein complex biogenesis | BP | 14 | 0.00491824 | 0.038170237 |
| GO:0006461 | protein complex assembly | BP | 14 | 0.00491824 | 0.038170237 |
| GO:1902531 | regulation of intracellular signal transduction | BP | 16 | 0.00492273 | 0.038170237 |
| GO:0031348 | negative regulation of defense response | BP | 4 | 0.00499675 | 0.038591457 |
| GO:0036474 | cell death in response to hydrogen peroxide | BP | 2 | 0.00501016 | 0.038591457 |
| GO:0000146 | microfilament motor activity | MF | 2 | 0.00501016 | 0.038591457 |
| GO:2000026 | regulation of multicellular organismal development | BP | 16 | 0.00503395 | 0.038689515 |
| GO:0043005 | neuron projection | CC | 11 | 0.00508507 | 0.038996659 |
| GO:1901565 | organonitrogen compound catabolic process | BP | 6 | 0.00510223 | 0.039042642 |
| GO:0043632 | modification-dependent macromolecule catabolic process | BP | 8 | 0.00525258 | 0.040105383 |
| GO:0044877 | macromolecular complex binding | MF | 13 | 0.00529318 | 0.040273586 |
| GO:0044772 | mitotic cell cycle phase transition | BP | 7 | 0.00529764 | 0.040273586 |
| GO:0006468 | protein phosphorylation | BP | 17 | 0.00540876 | 0.041029172 |
| GO:0016578 | histone deubiquitination | BP | 2 | 0.00544915 | 0.04124606 |
| GO:0022613 | ribonucleoprotein complex biogenesis | BP | 7 | 0.00561513 | 0.042410604 |
| GO:0005902 | microvillus | CC | 3 | 0.00567864 | 0.042797843 |
| GO:0010557 | positive regulation of macromolecule biosynthetic process | BP | 15 | 0.00571772 | 0.04299975 |
| GO:0042391 | regulation of membrane potential | BP | 6 | 0.0057774 | 0.043355281 |
| GO:0035295 | tube development | BP | 8 | 0.00586973 | 0.043900642 |
| GO:0071453 | cellular response to oxygen levels | BP | 4 | 0.00587518 | 0.043900642 |
| GO:0071824 | protein-DNA complex subunit organization | BP | 5 | 0.00600036 | 0.044682923 |
| GO:0051052 | regulation of DNA metabolic process | BP | 6 | 0.00601631 | 0.044682923 |
| GO:0032270 | positive regulation of cellular protein metabolic process | BP | 14 | 0.0060182 | 0.044682923 |
| GO:0042221 | response to chemical | BP | 30 | 0.00607832 | 0.045033634 |
| GO:1903362 | regulation of cellular protein catabolic process | BP | 5 | 0.00610099 | 0.04510605 |
| GO:0051051 | negative regulation of transport | BP | 7 | 0.00622229 | 0.045905781 |
| GO:0043901 | negative regulation of multi-organism process | BP | 4 | 0.00628118 | 0.046242706 |
| GO:0099536 | synaptic signaling | BP | 8 | 0.00635219 | 0.046374945 |
| GO:0098916 | anterograde trans-synaptic signaling | BP | 8 | 0.00635219 | 0.046374945 |
| GO:0007268 | chemical synaptic transmission | BP | 8 | 0.00635219 | 0.046374945 |
| GO:0099537 | trans-synaptic signaling | BP | 8 | 0.00635219 | 0.046374945 |
| GO:0042454 | ribonucleoside catabolic process | BP | 2 | 0.00637797 | 0.046466147 |
| GO:0051241 | negative regulation of multicellular organismal process | BP | 11 | 0.0064974 | 0.04723783 |
| GO:0006475 | internal protein amino acid acetylation | BP | 4 | 0.00656195 | 0.047608171 |
| GO:0030154 | cell differentiation | BP | 27 | 0.00663924 | 0.048069216 |
| GO:0016310 | phosphorylation | BP | 19 | 0.00670514 | 0.048446034 |
| GO:0034451 | centriolar satellite | CC | 2 | 0.00686746 | 0.049516533 |
| GO:0007155 | cell adhesion | BP | 14 | 0.00689584 | 0.049618817 |
