# Supplementary material for: Whole-genome sequencing of wild Siberian musk deer (Moschus moschiferus) provides insights into its genetic features
Source: BMC Genomics. 2020 Jan 31;21:108. doi: 10.1186/s12864-020-6495-2 (PMC6995116; doi:10.1186/s12864-020-6495-2)
Supplement: Supplementary file 3 — Additional file 3: Table S17. (Comparison of the genome assembly of Siberian musk deer and forest musk deer). [file 12864_2020_6495_MOESM3_ESM.docx]

**Supplementary Table S17. Comparison of the genome assembly of Siberian musk deer and forest musk deer.**

| **Assembly** | **Siberian musk deer** | **forest musk deer (Fan et al.)** |
| --- | --- | --- |
| Total length (Gb) | 2.70 | 2.72 |
| Contig N50 (Kb) | 29.1 | 22.6 |
| Scaffold N50 (Mb) | 7.95 | 2.85 |
| Scaffold number | 13,344 | 79,206 |
| Aligned length ^a^ (Gb) | 2.16 | 2.15 |
| 1-to-1 alignment length (Gb) | 2.13 | 2.13 |
| Average identity | 98.74% | 98.74% |

^a^ Only alignment blocks > 5Kb are considered.
